# Barriers and enablers to physical activity in patients during hospital stay: a scoping review

**DOI:** 10.1186/s13643-021-01843-x

**Published:** 2021-11-04

**Authors:** Sven Jacobus Gertruda Geelen, Hanneke Corine van Dijk - Huisman, Robert Adriaan de Bie, Cindy Veenhof, Raoul Engelbert, Marike van der Schaaf, Antoine François Lenssen

**Affiliations:** 1 Department of Rehabilitation Medicine, Amsterdam Movement Sciences, Amsterdam UMC, University of Amsterdam, Meibergdreef 9, Amsterdam, 1105AZ The Netherlands; 2 Department of Physical Therapy, Maastricht University Medical Centre, P. Debyelaan 25, Maastricht, 6229HX The Netherlands; 3 CAPHRI School for Public Health and Primary Care, Maastricht University, Maastricht, the Netherlands; 4 Department of Epidemiology, Maastricht University, Maastricht, the Netherlands; 5 Physical Therapy Research, Department of Rehabilitation, Physical Therapy Sciences & Sports, University Medical Centre Utrecht, Utrecht University, Utrecht, The Netherlands; 6 Expertise Centre Healthy Urban Living, Research Group Innovation of Human Movement Care, University of Applied Sciences Utrecht, Utrecht, The Netherlands; 7 Centre of Expertise Urban Vitality, Faculty of Health, Amsterdam University of Applied Sciences, Amsterdam, the Netherlands

**Keywords:** Physical activity, Mobility, Hospital, Barrier, Enabler, Theoretical domains framework

## Abstract

**Background:**

Low levels of physical activity are common during the hospital stay and have been associated with negative health outcomes. Understanding barriers and enablers to physical activity during a hospital stay can improve the development and implementation of tailored interventions aimed at improving physical activity. Previous studies have identified many barriers and enablers, but a comprehensive overview is lacking. This study aimed to identify and categorize all published patient- and healthcare professional-reported barriers and enablers to physical activity during a hospital stay for acute care, using the Theoretical Domains Framework (TDF).

**Methods:**

We conducted a scoping review of Dutch and English articles using MEDLINE, CINAHL Plus, EMBASE, PsycINFO, and Cochrane Library (inception to September 2020), which included quantitative, qualitative, and mixed-methods studies reporting barriers and enablers to physical activity during a hospital stay for acute care, as perceived by patients or healthcare professionals. Two reviewers systematically extracted, coded, and categorized all barriers and enablers into TDF domains.

**Results:**

Fifty-six articles were included in this review (32 qualitative, 7 quantitative, and 17 mixed-methods). In total, 264 barriers and 228 enablers were reported by patients, and 415 barriers and 409 enablers by healthcare professionals. Patient-reported barriers were most frequently assigned to the TDF domains *Environmental Context & Resources* (*ECR, n* = 148), *Social Influences* (*n* = 32), and *Beliefs about Consequences* (*n* = 25), while most enablers were assigned to *ECR* (*n* = 67), *Social Influences* (*n* = 54), and *Goals* (*n* = 32). Barriers reported by healthcare professionals were most frequently assigned to *ECR* (*n* = 210), *Memory, Attention and Decision Process* (*n* = 45), and *Social/Professional Role & Identity* (*n* = 31), while most healthcare professional-reported enablers were assigned to the TDF domains *ECR* (*n* = 143), *Social Influences* (*n* = 76), and *Behavioural Regulation* (*n* = 54).

**Conclusions:**

Our scoping review presents a comprehensive overview of all barriers and enablers to physical activity during a hospital stay and highlights the prominent role of the TDF domains *ECR* and *Social Influences* in hospitalized patients’ physical activity behavior. This TDF-based overview provides a theoretical foundation to guide clinicians and researchers in future intervention development and implementation.

**Scoping review registration:**

No protocol was registered for this review.

**Supplementary Information:**

The online version contains supplementary material available at 10.1186/s13643-021-01843-x.

## Contributions to the literature

- Physical inactivity during the hospital stay is a frequent problem, but an overview of patient- and healthcare professional-reported barriers and enablers to physical activity was lacking.
- The majority of barriers and enablers were categorized under the TDF-domains *Environmental Context and Resources* and *Social Influences*, highlighting the need for interventions that target the physical environment, hospital care processes, organizational characteristics, resources, patient-related factors, and social influences.
- Our comprehensive theory-informed overview of all published barriers and enablers to physical activity during a hospital stay can assist clinicians and researchers in developing and implementing tailored interventions in local clinical practice.

## Background

Hospitalized patients spend between 87 and 100% of their time lying in bed or sitting, irrespective of the reason for admission [1]. Low levels of physical activity have been associated with negative health outcomes like functional decline [2, 3], increased length of stay [4], increased risk of institutionalization [5, 6], and mortality [2, 3, 7, 8]. Previous research has shown that these negative health outcomes of inactivity can be counteracted by increasing physical activity levels [9–13]. Thus, interventions aimed at increasing the physical activity levels of hospitalized patients are of great importance [14].

Many different barriers and enablers influence patients’ physical activity behavior [14–20]. While barriers reduce or negatively affect a patient’s physical activity behavior [15, 18, 21], enablers enhance or positively affect this behavior [14, 16, 19, 20]. Brown et al. have investigated barriers to physical activity in older adults admitted to a medical ward [15]. They identified having symptoms (e.g., weakness, pain, fatigue), being concerned about falls, and a lack of staff to assist with out-of-bed physical activity as frequently reported barriers. So et al. also described not being provided with adequate walking aids and being attached to an intravenous line as barriers [14]. Moreover, they identified many enablers, such as being encouraged to exercise, preventing the negative effects of prolonged bed rest, and promoting functional recovery.

Over the past two decades, the number of studies identifying barriers or enablers to physical activity during a hospital stay for acute care has grown significantly [14–21]. In these studies, barriers and enablers were identified in a wide variety of patient populations and clinical settings [14–21]. Furthermore, they were explored from the perspective of patients [14, 20], healthcare professionals (HCPs) [16–18, 21], or both [15, 19]. To our knowledge, no comprehensive overview of barriers and enablers to physical activity during a hospital stay for acute care has been published. Such a comprehensive overview would provide clinicians and researchers with a better understanding of these barriers and enablers. This might improve the development of future interventions or implementation of existing interventions in different health care settings.

To be able to use such an overview in future intervention development or translation, it is essential to adopt a theoretical framework that links barriers and enablers to intervention strategies. A theoretical framework can help to guide interventions targeting modifiable factors for physical activity during the hospital stay for acute care [22, 23]. Moreover, using a theoretical framework to identify barriers and enablers to behavioral change has been demonstrated to be more successful in changing behavior than using a non-theory-driven approach [24, 25].

One such integrative theoretical framework is the Theoretical Domains Framework (TDF) [25]. The TDF facilitates a systematic and theoretically based approach to behavior change. The validated TDF contains 14 domains, comprising 84 theoretical constructs from 33 theories of behavior and behavior change. Barriers and enablers can be categorized in the following domains: *Knowledge*, *Skills*, *Social/Professional Role and Identity (SPRI)*, *Beliefs About Capabilities*, *Optimism*, *Beliefs about Consequences*, *Reinforcement*; *Intentions*, *Goals*, *Memory, Attention and Decision Processes (MADP)*, *Environmental Context and Resources (ECR)*, *Social Influences*, *Emotion*, and *Behavioural Regulation*. The TDF has been extensively used as a guide to identify and categorize modifiable factors that influence behavior [25]. The objective of this review was to identify and categorize patient- and HCP-reported barriers and enablers to physical activity during a hospital stay for acute care, using the TDF.

## Methods

### Study design

A scoping review was performed to explore the nature and quantity of published literature on barriers and enablers to physical activity during a hospital stay for acute care, as perceived by hospitalized patients and their HCPs. We used the scoping review methodology suggested by Arksey and O’Malley [26] and developed further by Levac, Colquhoun, and O’Brien [27, 28]. The Joanna Briggs Institute (JBI) guidance document for the conduct of scoping reviews and the Preferred Reporting Items for Systematic Reviews and Meta-Analyses Extension for Scoping Review (PRISMA-ScR) were used to inform the methodology (Additional file 1) [29, 30]. The TDF was used to categorize the barriers and enablers extracted from the included studies [25], as described in further detail in “Collating, summarizing, and reporting the results”. No protocol was registered for this review.

### Search strategy and study selection

A comprehensive search strategy was developed in collaboration with an experienced research librarian (FvE) of the University of Amsterdam (Additional file 2). An electronic database search of MEDLINE (through Pubmed), CINAHL Plus, Cochrane, EMBASE, PsycINFO, and the Cochrane library was performed, from the inception of the electronic databases to September 23, 2020.

All electronic database searches were combined and de-duplicated in Endnote version X9.1 (Clarivate Analytics, Philadelphia, Pennsylvania, USA) [31]. Two reviewers (SJGG and HCvDH) independently screened all titles and abstracts to determine eligibility, based on the following inclusion and exclusion criteria. Studies were considered eligible if they reported barriers or enablers to physical activity during a hospital stay as perceived by patients or HCPs. Patients had to be hospitalized in an acute care setting and HCPs had to be involved in clinical care (e.g., physicians, nurses, nursing assistants, occupational therapists, and physiotherapists). Barriers were defined as any factor reducing or negatively affecting a patient’s engagement in physical activity. Enablers were defined as any factor enhancing or positively affecting a patient’s engagement in physical activity. Barriers and enablers had to be self-reported. Studies reporting factors associated or correlated to physical activity during the hospital stay for acute care that was not self-reported were not included in this study [32]. Published full-text articles using quantitative, qualitative, or mixed-method study designs were considered, as was gray literature (i.e., academic papers, theses, and dissertations). Only studies written in English or Dutch were included. Studies reporting solely on children (<18 years), short-stay admissions (<24 h), the Intensive Care Unit, or psychiatric ward were excluded because of the differences in care and context (e.g., in terms of organization of care, length of hospital stay, patient characteristics, and care provided). Protocols and reviews were excluded as they lack empirical data. Case studies were also excluded as they often describe extreme cases that do not represent the general population of hospitalized patients. Lastly, conference abstracts were excluded.

To ensure that at least 80% agreement was reached between the reviewers in determining eligibility based on study titles and abstracts, a pilot was performed using 5% of the references. The pilot resulted in minor revisions of the inclusion and exclusion criteria, to enhance the clarity of the criterion descriptors. Full-text articles were obtained when studies fulfilled the criteria or when additional information was needed to determine eligibility. Subsequently, full-text articles were independently screened by both reviewers to determine eligibility. To ensure that at least 80% agreement was reached between reviewers in determining eligibility based on full texts, a pilot was first performed using 10% of the references.

To reduce the risk of missing relevant studies, reference lists of included studies and the reviewers’ own literature databases were screened for additional studies. Any disagreements during the study selection process were resolved by discussion, mediated by a senior researcher (AFL). The web application of Rayyan QCRI (Qatar Computing Research Institute, Hamad Bin Khalifa University) was used to facilitate the study selection process [33]. A PRISMA-ScR flowchart was created to track the screening and inclusion process of this review [30, 33].

### Data extraction

Both reviewers (SJGG and HCvDH) independently extracted data using a custom-built data extraction form. Characteristics of included studies (author(s), year of publication, type of study, study aim, method, population, setting, and study sample) were extracted according to the JBI Guidance document for the conduct of scoping reviews [29]. Barriers and enablers identified in the results sections of the included studies were extracted using an iterative data extraction process. Barriers and enablers reported by patients and HCPs were extracted separately. Different extraction methods were used for qualitative and quantitative studies [34]. From qualitative studies, all barriers and enablers reported by patients or HCPs were extracted. For quantitative studies, the approach described by Weatherson [35] was used, meaning that barriers and enablers were extracted if ≥50% of participants agreed that the factor influenced patients’ physical activity behavior. For example, in a survey with dichotomous answering options (agree/disagree), the factor “discussing physical activity during physician rounds increases patients’ physical activity levels” was not extracted as an enabler if 42% of the HCPs agreed. Some questionnaire measures contained an intermediate category, such as 5-point Likert-scale questions with answering options: 1 = strongly agree, 2 = somewhat agree, 3 = neither agree nor disagree, 4 = somewhat disagree, and 5 = strongly disagree. Barriers or enablers were only extracted if at least 50% of participants somewhat agreed or strongly agreed that they perceived it as a barrier or enabler [35]. For example, if 60% of the HCPs agreed (18% somewhat agreed and 42% strongly agreed) that “discussing physical activity during physician rounds increases patients’ physical activity levels” was an enabler, this factor was extracted as enabler [35]. If a quantitative study included open-ended questions, the responses were extracted as in qualitative studies.

To ensure the reliability of the data extraction process, the reviewers first extracted data from five randomly selected articles [14–16, 19, 36] and discussed their findings to resolve disagreements and improve the preliminary data extraction table. This process was then repeated with five other articles [17, 21, 37–39], after which both researchers agreed on the data extraction and no further changes to the data extraction table were required. Finally, each reviewer independently extracted half of the remaining articles and then critically reviewed the extraction of the other half performed by the other reviewer. Disagreements were resolved by discussion and rereading source material, and two senior researchers were consulted in case of discrepancies (AFL and MvdS).

### Collating, summarizing, and reporting the results

Both reviewers (SJGG and HCvDH) independently coded the extracted barriers and enablers and categorized them into the 14 TDF domains [25, 40, 41]. The theoretical definitions and component constructs of the domains as presented in Additional file 3 were used to guide the coding process. Barriers and enablers were coded separately for patients and HCPs and were coded to more than one domain if the content suited multiple domains. To increase inter-coder reliability, the two reviewers (SJGG and HCvDH) met to discuss coding discrepancies and to iteratively modify the coding structure after every ten articles. Discrepancies were solved by discussion and rereading the articles. If necessary, a senior researcher (MvdS) was consulted to discuss and resolve discrepancies. This process was repeated until a final TDF categorization had been obtained. Two senior researchers (AFL and MvdS) supervised the categorization process. The entire authorship team reviewed the final categorization. MAXQDA Analytics Plus 2020 (VERBI Software, 2018, Berlin, Germany) was used to facilitate data coding and the categorization process. The numbers of different barriers and enablers assigned per TDF domain as well as the number of articles reporting on barriers and enablers per TDF domain were presented separately for patients and HCPs. Finally, a descriptive summary of the reported barriers and enablers was composed for patients and HCPs.

## Results

**Fig. 1:**
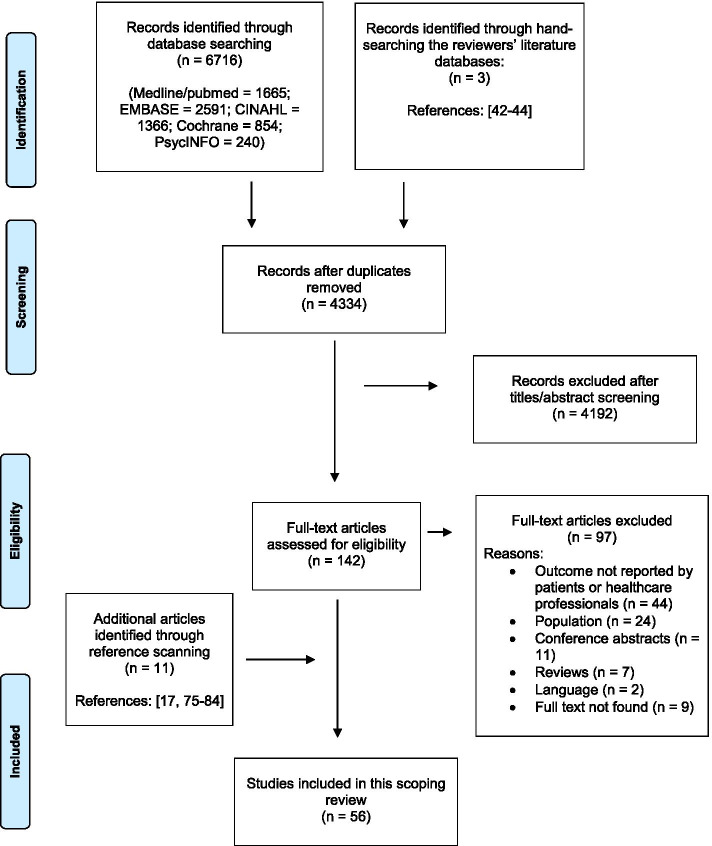
PRISMA-ScR flowchart

The search retrieved 6716 studies, of which 2382 were excluded as duplicates. An additional three studies [42–44] were retrieved by hand-searching the researchers’ own literature database (i.e., two studies which did not explicitly mention “barrier,” “enabler,” or “hospital” in the title and abstract, and one which was a Masters thesis). A total of 4334 studies were screened based on titles and abstracts. Of the 143 articles that were assessed as full texts, 45 were identified for inclusion [11, 14–16, 18–21, 36–39, 42–74]. An additional 11 studies were included after hand-searching the reference lists of included studies [17, 75–84], resulting in a total of 56 included studies [11, 14–21, 36–39, 42–84]. The PRISMA-ScR flowchart (Fig. 1) shows the screening and inclusion process.

### Description of included studies

Additional file 4 presents an overview of the included studies. Of the 56 studies, 32 used a qualitative study design [14–20, 38, 42, 44–46, 49–54, 57, 61, 67, 68, 70, 72–76, 78–80, 83], seven a quantitative study design [21, 37, 39, 63, 66, 69, 82], and 17 a mixed-methods study design [11, 36, 43, 47, 48, 55, 56, 58–60, 62, 64, 65, 71, 77, 81, 84]. Nineteen studies reported barriers and enablers as perceived by patients [14, 20, 36, 37, 39, 48, 51, 61, 63–65, 67, 70, 73, 78, 79, 81, 83], 23 reported those perceived by HCPs [16–18, 21, 38, 42, 43, 47, 50, 52–55, 57, 59, 60, 68, 69, 74–76, 80, 82], and 14 reported those perceived by patients and HCPs [11, 15, 19, 44–46, 49, 56, 58, 62, 66, 71, 72, 77, 84]. Sample sizes varied between *n* = 6 and *n* = 345 patients and between *n* = 5 and *n* = 261 HCPs. Two studies did not specify the sample size [11, 77], and one study only specified the number of included sites [47]. Further descriptions of the populations and settings included are provided in Additional file 4. The included studies were published between 2003 and 2020, and only seven studies were published before 2010 [15, 52, 56, 75, 77, 78, 82].

### Identification of patient- and HCP-reported barriers and enablers to physical activity during a hospital stay for acute care

The results of the data extraction process are presented in Additional file 5. After coding and discussing all extracted fragments containing barriers and enablers, SJGG and HCvDH reached a consensus on 1316 barriers and enablers. Two hundred sixty-four (20.2%) patient-reported barriers and 415 (31.7%) HCP-reported barriers were coded. Two hundred twenty-eight (17.3%) patient-reported enablers and 409 (31.2%) HCP-reported enablers were coded.

### Categorizing patient- and HCP-reported barriers using the TDF

**Fig. 2:**
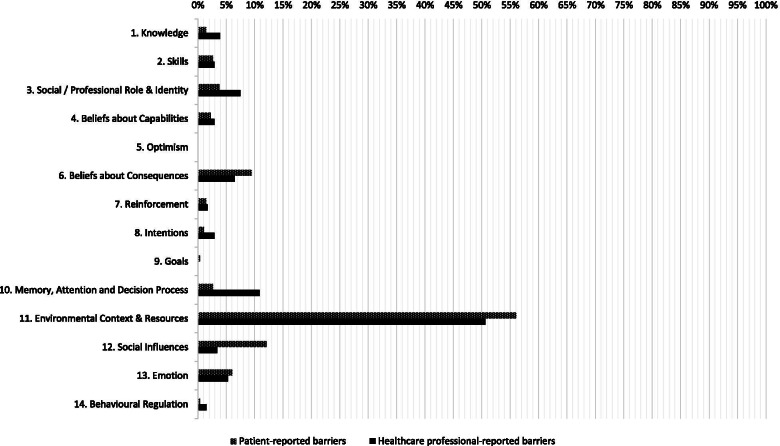
Barriers assigned to each domain of the Theoretical Domains Framework (% of the total number of reported barriers)

Patient- and HCP-reported barriers were assigned to 13 of the 14 TDF domains. An overview of the TDF coding of all barriers is provided in Additional file 6 and summarized in Fig. 2. Patient-reported barriers were assigned most frequently to the TDF domains *ECR* (*n* = 148, 56.1%), *Social Influences* (*n* = 32, 12.1%), and *Beliefs about Consequences* (*n* = 25, 9.5%). Of the other 11 domains, the largest numbers of barriers were assigned to the domains *Emotion* (*n* = 16, 6.1%) and *SPRI (n* = 10, 3.8%). HCP-reported barriers were assigned most frequently to the TDF domains *ECR* (*n* = 210, 50.6%), *MADP* (*n* = 45, 10.8%), and *SPRI* (*n* = 31, 7.5%). Of the other 11 domains, the largest numbers of barriers were assigned to the domains *Beliefs about Consequences* (*n* = 27, 6.5%) and *Emotion* (*n* = 22, 5.3%)*.* No patient- and HCP-reported barriers were assigned to the domain *Optimism.*

The TDF domains to which barriers were most frequently assigned are highlighted below. The domain *ECR* had the majority of both patient- and HCP-reported barriers assigned to it and covered four main topics: (1) patient-related factors (e.g., medical factors, age, language barriers), (2) care processes and organizational characteristics (e.g., prescribed immobility, communication, hospital culture, bed-centered care), (3) physical environment of the hospital (e.g., room, unit, hospital), and (4) resources (e.g., limited time, staffing, equipment) (Additional file 6). Patient-reported barriers assigned to the domain *Social Influences* included interpersonal processes between patients, visitors, and HCPs that negatively influence physical activity, such as lack of encouragement and assistance and providing more care than necessary. Patient-reported barriers assigned to the domain *Beliefs about Consequences* included the belief that physical activity results in negative consequences (e.g., injuries, falling, or missing meals and care), the belief that rest is needed for recovery, and the belief that patients may be inconveniencing busy HCPs. Most of the HCP-reported barriers assigned to the domain *MADP* related to prioritization. A high workload and safety considerations resulted in physical activity receiving a lower priority than medical treatment or rest. HCP-reported barriers assigned to the domain *SPRI* included the passive and dependent attitude patients adopt during hospitalization (e.g., the idea that patients should remain in bed, personality, and character traits). In addition, HCPs mentioned the role they fulfill regarding physical activity (e.g., lack of role clarity in improving physical activity, attributing responsibility to others, and nurses lacking autonomy in deciding how and when to mobilize patients).

### Categorizing patient- and HCP-reported enablers using the TDF

**Fig. 3:**
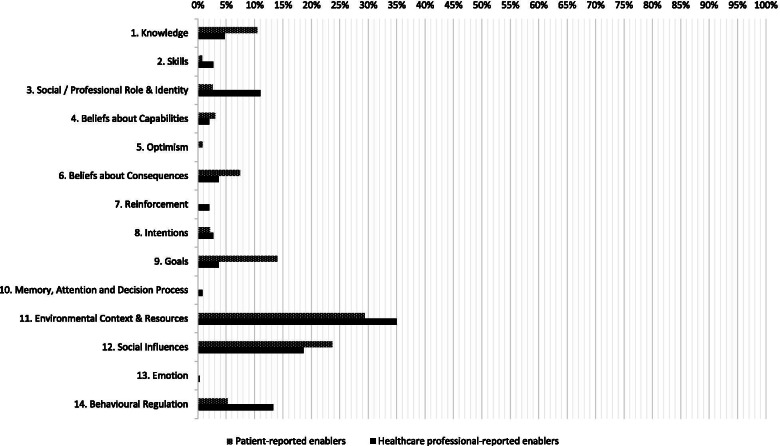
Enablers assigned to each domain of the Theoretical Domains Framework (% of the total number of reported enablers)

Patient- and HCP-reported enablers were assigned to 11 and 13 of the 14 TDF domains, respectively. An overview of the TDF-coding of all enablers is provided in Additional file 7 and summarized in Fig. 3. Patient-reported enablers were most frequently assigned to the TDF domains *ECR* (*n* = 67, 30.2%), *Social Influences* (*n* = 54, 24.3%), and *Goals* (*n* = 32, 14.4%). Of the remaining 11 domains, the largest numbers of enablers were assigned to the domains *Knowledge* (*n* = 24, 10.5%) and *Beliefs about Consequences* (*n* = 17, 7.7%)*.* No patient-reported enablers were assigned to the domains *Reinforcement, MADP*, and *Emotion.* HCP-reported enablers were most frequently assigned to the TDF domains *ECR* (*n* = 143, 35.0%), *Social Influences* (*n* = 76, 18.6%), and *Behavioral Regulation* (*n* = 54, 13.2%). Of the remaining 11 domains, the largest numbers of enablers were assigned to the domains *SPRI* (*n* = 45, 11%) and *Knowledge* (*n* = 19, 4.7%). No HCP-reported enablers were assigned to the domain *Optimism*.

In line with the categorization of the barriers, most patient- and HCP-reported enablers were assigned to the domain *ECR* and covered the same four main topics: (1) patient-related factors, (2) care processes and organizational characteristics, (3) physical environment of the hospital, and (4) resources (Additional file 7). Patient- and HCP-reported enablers assigned to the domain *Social Influences* included interpersonal processes between patients and visitors or HCPs that positively influence physical activity, such as being encouraged and assisted. Patients also described that other patients motivated them to perform more physical activity, while HCPs described how leadership and multidisciplinary collaboration enabled them to improve patients’ physical activity. Patient-reported enablers assigned to the domain *Goals* included the importance of having a goal (e.g., experiencing the positive effects of physical activity or preventing the negative effects of physical inactivity). This domain also included the importance of having autonomy and being involved in physical activity-related decision-making. HCP-reported enablers assigned to the domain *Behavioural Regulation* included strategies aimed at regulating behavior, such as providing education, appointing mobility champions, making performance and expectations visible, creating a habit, and using mobility documentation tools, reminders, daily schedules, exercise programs, and mobility audits.

## Discussion

The aim of this study was to identify and categorize patient- and HCP-reported barriers and enablers to physical activity during a hospital stay for acute care, using the TDF. Our systematic search identified 679 barriers and 637 enablers, reported in 56 studies. The majority of barriers and enablers were assigned to the key domain *Environmental Context and Resources* (i.e., “patient-related factors,” “care processes and organizational characteristics,” “physical environment of the hospital,” and “resources”). Other key TDF domains to which the largest numbers of barriers were assigned were *Social Influences*, *Beliefs about Consequences*, *Memory, Attention and Decision Process,* and *Social/Professional Role & Identity*. Additionally, other key domains to which the largest numbers of enablers were assigned were *Social Influences*, *Goals, and Behavioural Regulation*. This is the first scoping review of patient- and HCP-reported barriers and enablers relating to physical activity during the hospital stay for acute care using a TDF analysis. This review presents a comprehensive overview of these barriers and enablers from a theoretical perspective, which can help clinicians and researchers identify and target modifiable factors within future intervention development.

Our findings highlight the prominent role of the domain *Environmental Context and Resources* with respect to physical activity during the hospital stay for acute care. Upon hospital admission, patients are taken out of their own environment and enter a different, unfamiliar context, filled with many uncertainties. In addition to patients’ illness and associated medical factors, the hospital environment exerts an inactivating influence on patients, resulting in a loss of autonomy and freedom [15, 44]. Our findings indicate that “care processes and organizational characteristics,” the “physical environment,” “patient-related factors,” and “resources” are the main topics of the domain *Environmental Context and Resources* that influence the physical activity behavior of hospitalized patients. Several studies have aimed to improve physical activity in hospitalized patients by targeting these main environmental factors [11, 12, 71, 85]. “Care processes and organizational characteristics” was targeted by incorporating physical activity in usual care [12, 85, 86], creating policy to promote mobility [71], incorporating specific timeslots for physical activity in HCPs’ schedules [71], improving communication [12], and providing patients with graded exercise programs [11]. “Physical environment” was targeted by providing interesting walking destinations [11], marked walking trails [71], distance markers in the hallway [71], ward maps and signs [11], and by making mobilization goals visible [12, 86]. “Patient-related factors” were targeted by optimizing pain control [12], and “resources” by purchasing more walking aids [71], supporting physical activity with technology [86, 87], and supplying activity diaries and exercise booklets [11, 85].

Our results also highlight the role of the domain *Social Influences*, identified as the second most prominent TDF domain. The absence of encouragement and assistance by others (i.e., nurses, physical therapists, physicians, visitors, volunteers, or other patients) was identified as an important barrier by patients, whereas their presence as an important enabler. This was substantiated by HCPs, who also added multidisciplinary teamwork, leadership support, the presence of physical therapists, and involving visitors as important enablers of physical activity. Several studies have aimed to improve physical activity by targeting the domain *Social Influences*, by providing systematic encouragement and assistance from HCPs [11, 71, 85, 86], involving volunteers or family members in basic mobility activities [11, 86], and encouraging independence in activities of daily living [11].

Moreover, the domains *Beliefs about Consequences*, *Memory, Attention and Decision Process*, and *Social/Professional Role & Identity* also contained many barriers. Several studies have targeted these domains to improve patients’ physical activity levels, such as providing education to counter the belief that physical activity will result in injuries [86, 88], using shift huddles to address prioritizing physical activity [89], or mapping the therapy consultation process within a multidisciplinary team to create role clarity and avoid unnecessary treatments [90]. Likewise, the domains *Goals* and *Behavioural Regulation* contained many enablers. Examples of interventions that specifically focus on goal setting and behavioral regulation are the Johns Hopkins Highest Level of Mobility tool [12], the WALK-FOR 900 steps per day behavioral target [91], and Hospital Fit monitor [87]. All these interventions enable monitoring physical activity levels and setting physical activity goals in daily clinical care.

Our findings indicated that there were several TDF domains (e.g., *Skills*, *Optimism*, *Reinforcement*) to which few or no barriers and enablers were assigned. The many factors assigned to the TDF domains *Environmental Context and Resources* and *Social Influences*, and the few factors assigned to the domains *Skills, Optimism,* and *Reinforcement* are in agreement with the results of similar research performed in other populations, such as physical activity at school [35], work [92], or in primary care [93]. Although this highlights the prominent role of the domains *Environmental Context and Resources* and *Social Influences* on physical activity behavior, it does not indicate whether the domains *Skills, Optimism,* and *Reinforcement* do not contain relevant barriers and enablers to physical activity, or whether they were under-identified.

Lastly, although many patient-reported barriers and enablers were also reported by HCPs, our results demonstrated that HCPs perceived a greater number of barriers and enablers than patients. This could be explained by the different perspectives of patients and HCPs on physical activity during the hospital stay. Patients are hospitalized for a relatively short period, with their main focus being their illness and getting better. They experience how it feels to be a patient and how this influences their physical activity behavior. On the other hand, HCPs perceive barriers and enablers from a much broader perspective. Firstly, they report barriers and enablers from their own as well as their patients’ perspectives. Secondly, they provide care to many patients with different pathologies, ages, and backgrounds. Thirdly, they perceive barriers and enablers related to providing care, different care processes, and organizational characteristics. These differences in perspectives between patients and HCPs emphasize that both must be taken into account to gain a comprehensive understanding of the barriers and enablers to physical activity during a hospital stay.

### Strengths and limitations

This is the first scoping review on patient- and HCP-reported barriers and enablers relating to physical activity during the hospital stay for acute care using a TDF analysis. A strength of this study is that it was designed and conducted according to the systematic scoping review methodology and that it followed the PRISMA-ScR statement recommendations [26–30]. Secondly, almost all aspects of data collection, data extraction, and data analysis were carried out independently by two researchers, with a third party available in case of disagreements. Thirdly, given the extensive, thorough search strategy in multiple databases, along with the inclusion of quantitative, qualitative as well as mixed-methods study designs, we were able to present a complete overview of all barriers and enablers reported in the current literature. Fourthly, an additional strength of this study is the use of the TDF as a theoretical framework to categorize barriers and enablers. The use of the TDF ensured that the reviewers assessed barriers and enablers from a broad perspective, thereby also exploring underexposed domains.

We also recognize some limitations. While the use of the TDF facilitates reviewers in exploring barriers and enablers from a broad perspective, it does not provide an explanation as to how barriers and enablers are connected and influence one another. Another limitation of this study is that barriers and enablers are presented based on the number of articles in which they have been reported. As the frequency of reporting is primarily a function of the methods used to present the data, this alone should not be used as a proxy of importance. In other words, a barrier that has only been reported once may be just as relevant as one that has been reported many times. Furthermore, a secondary analysis of differences in perceived barriers and enablers among patient subgroups or among professions could not be performed due to the lack of detailed reporting in the included studies. Lastly, as this was a scoping review, no quality appraisal of included articles was performed [30].

### Clinical implications and recommendations for future research

Our findings provide a comprehensive overview of barriers and enablers to physical activity during a hospital stay for acute care. The large number of barriers and enablers we found, distributed across many TDF domains, highlight the complexity of physical activity behavior during the hospital stay and the need for tailored interventions. A context-based assessment should be performed to determine which barriers and enablers can be targeted in a specific clinical setting. Our comprehensive overview will enable clinicians and researchers to perform this context-based assessment from a broad perspective and support them in establishing a behavioral diagnosis of what needs to change in a specific context in order to improve physical activity behavior during the hospital stay.

Subsequently, clinicians and researchers will be able to link relevant barriers and enablers to specific intervention strategies and behavior change techniques (BCTs) [25, 41, 94]. An example of a framework that could be used to assist clinicians and researchers in selecting appropriate BCTs is the Behaviour Change Wheel (BCW) [22]. Our TDF-based overview provides the initial step in developing and implementing theory-informed behavior change interventions aimed at improving physical activity during the hospital stay [41].

Given the large number of factors influencing the physical activity behavior of hospitalized patients, we recommend that clinicians and researchers develop and implement interventions targeted at multiple barriers and enablers. Previous research suggests that developing and implementing such tailored multimodal interventions may be more effective than unimodal interventions [95]. Moreover, given a large number of barriers and enablers assigned to the *Environmental Context and Resources* and *Social Influences* context in our review, we suggest that clinicians and researchers should always consider incorporating intervention strategies targeting these TDF domains in their multimodal interventions.

Future research should focus on exploring relationships between barriers and enablers both within and between TDF domains. Revealing these relationships may facilitate the assessment of barriers and enablers in specific clinical settings and may increase the effectivity of future tailored multimodal interventions. Future research is also needed to explore the differences in perspectives perceived by different patient subgroups (e.g., age, sex, pathologies). Similarly, more research is needed to investigate differences in perceived barriers and enablers among professions and how these differences relate to their role in improving physical activity during the hospital stay. Additionally, further research is needed to develop and validate a TDF-based questionnaire that could facilitate the context-based assessment of barriers and enablers across all TDF domains. Further research is needed to retrospectively identify which barriers and enablers to physical activity during the a hospital stay have been targeted in previously described intervention studies [94], so clinicians may be better able to implement these interventions in other contexts. Finally, there is a need for research assessing the effectiveness of tailored multimodal interventions that target context-based barriers and enablers to physical activity in hospitalized patients.

## Conclusions

This article presents a comprehensive overview of 1316 patient- and HCP-reported barriers and enablers to physical activity during a hospital stay for acute care. A large number of barriers and enablers found highlight the complexity of physical activity behavior during the hospital stay. Our overview can assist clinicians and researchers in performing a context-based assessment to determine which barriers and enablers to target in future interventions. Given the large number of factors influencing the physical activity behavior of hospitalized patients, we recommend developing and implementing multimodal interventions. This scoping review also highlights the large role of environmental and social factors on physical activity behavior during the hospital stay and suggests that intervention strategies targeting these domains should be incorporated. Future research should focus on exploring the relationships between barriers and enablers both within and between different TDF domains. Revealing these relationships may facilitate the assessment of barriers and enablers in specific clinical settings and may increase the effectivity of future tailored multimodal interventions. Furthermore, future research is also needed to explore the differences in perspectives perceived among different patient subgroups or different professions. Lastly, a validated TDF-based questionnaire is needed to facilitate future context-based assessments of barriers and enablers, and further research should investigate the effectiveness of tailored multimodal interventions.

## Supplementary Information

**Additional file 1.** PRISMA-ScR checklist. Completed PRISMA-ScR checklist for this scoping review.

**Additional file 2.** Search terms. Presentation of the used search terms with databases.

**Additional file 3.** The Theoretical Domains Framework with definitions and component constructs. Description of the definitions and component constructs of each TDF domain.

**Additional file 4.** Characteristics of included studies. Table presenting the characteristics of included studies.

**Additional file 5.** Data extraction. Excel file presenting all extracted data from included studies.

**Additional file 6.** Barriers to physical activity during hospital stay for acute care as reported by patients and healthcare professionals. Table presenting the barriers to physical activity.

**Additional file 7.** Enablers to physical activity during a hospital stay for acute care as reported by patients and healthcare professionals. Table presenting the enablers to physical activity.

## Data Availability

The datasets used and/or analyzed during the current study are available from the corresponding author on reasonable request.

## Abbreviations

HCPs: Healthcare professionals
JBI: Joanna Briggs Institute
PRISMA-ScR: Preferred Reporting Items for Systematic Reviews and Meta-Analyses Extension for Scoping Reviews
TDF: Theoretical Domains Framework
UK: United Kingdom
USA: United States of America
