# Supplementary material for: Barriers and enablers to physical activity in patients during hospital stay: a scoping review
Source: Syst Rev. 2021 Nov 4;10:293. doi: 10.1186/s13643-021-01843-x (PMC8569983; doi:10.1186/s13643-021-01843-x)
Supplement: Supplementary file 2 — Additional file 2. Search terms. Presentation of the used search terms with databases. [file 13643_2021_1843_MOESM2_ESM.pdf]

Additional File 2. Search terms

The search strategy included the following terms and concepts: ‘hospitalization’, ‘physical activity’, ‘mobility’, ‘ambulation’, ‘walking’, ‘barrier’, ‘enabler’, ‘motivator’, and ‘facilitator’. This resulted in the following search strings:

<u>Pubmed (MEDLINE)</u>

("Hospitalization"[Mesh] OR "Inpatients"[Mesh] OR "Adolescent, Hospitalized"[Mesh] OR "Hospital Units"[Mesh] OR hospitalization[tiab] OR hospitalisation[tiab] OR hospitalized[tiab] OR hospitalised[tiab] OR hospital environment[tiab] OR hospital stay*[tiab] OR hospital setting*[tiab] OR medical ward*[tiab])

AND

("Motor Activity"[Mesh] OR "Early Ambulation"[Mesh] OR physical activit*[tiab] OR physical function*[tiab] OR mobility[tiab] OR mobilis*[tiab] OR mobiliz*[tiab] OR exercis*[tiab] OR motor activit*[tiab] OR walking[tiab] OR ambulat*[tiab])

AND

(barrier*[tiab] OR enabl*[tiab] OR facilitat*[tiab] OR motivat*[tiab])

AND

(english[Language] OR dutch[Language])

NOT

(("Animals"[Mesh] OR "Invertebrates"[Mesh] OR "Plants"[Mesh] OR "Fungi"[Mesh] OR "Animal Experimentation"[Mesh] OR "Models, Animal"[Mesh] OR animal experiment*[tiab] OR animal model*[tiab]) NOT "Humans"[Mesh])

<u>Embase (ovid)</u>

#	Searches
1	hospitalization/ or exp hospital patient/ or exp *"hospital subdivisions and components"/ or (hospitalization or hospitalisation or hospitalized or hospitalised or hospital environment or hospital stay* or hospital setting* or medical ward*).ti,ab,kw.
2	exp motor activity/ or mobilization/ or (physical activit* or physical function* or mobility or mobilis* or mobiliz* or exercis* or motor activit* or walking or ambulat*).ti,ab,kw.

3	(barrier* or enabl* or facilitat* or motivat*).ti,ab,kw.
4	1 and 2 and 3
5	limit 4 to (dutch or english)
6	(exp animal/ or exp invertebrate/ or exp plant/ or exp fungus/ or exp animal experiment/ or exp animal model/ or (animal experiment* or animal model*).ti,ab,kw.) not human/
7	5 not 6
8	limit 7 to conference abstract status
9	7 not 8

PscycINFO (ovid)

#	Searches
1	exp hospitalization/ or hospitalized patients/ or (hospitalization or hospitalisation or hospitalized or hospitalised or hospital environment or hospital stay* or hospital setting* or medical ward*).ti,ab,id.
2	exp physical activity/ or (physical activit* or physical function* or mobility or mobilis* or mobiliz* or exercis* or motor activit* or walking or ambulat*).ti,ab,id.
3	(barrier* or enabl* or facilitat* or motivat*).ti,ab,id.
4	1 and 2 and 3
5	limit 4 to (dutch or english)

Cochrane Library

ID Search

#1 MeSH descriptor: [Inpatients] explode all trees

#2 MeSH descriptor: [Adolescent, Hospitalized] explode all trees

#3 MeSH descriptor: [Hospital Units] explode all trees

#4 (hospitalization or hospitalisation or hospitalized or hospitalised or hospital environment or hospital stay* or hospital setting* or medical ward*):ti,ab,kw

#5 #1 or #2 or #3 or #4

#6 MeSH descriptor: [Motor Activity] explode all trees

#7 MeSH descriptor: [Early Ambulation] explode all trees

#8 (physical activit* or physical function* or mobility or mobilis* or mobiliz* or exercis* or motor activit* or walking or ambulat*):ti,ab,kw

#9 #6 or #7 or #8

#10 (barrier* or enabl* or facilitat* or motivat*):ti,ab,kw

#11 #5 and #9 and #10 in Cochrane Reviews, Trials

#12 ((clinicaltrials or trialsearch)):so

#13 #11 not #12

<u>CINAHL (Ebsco):</u>

Language: Dutch/Flemish, English

(MH "Hospitalization+") OR (MH "Inpatients") OR (MH "Adolescent, Hospitalized") OR (MH "Hospital Units+") OR (TI (hospitalization or hospitalisation or hospitalized or hospitalised or hospital environment or hospital stay* or hospital setting* or medical ward*) OR AB (hospitalization or hospitalisation or hospitalized or hospitalised or hospital environment or hospital stay* or hospital setting* or medical ward*))

AND

(MH "Motor Activity+") OR (MH "Early Ambulation") OR (TI (physical activit* or physical function* or mobility or mobilis* or mobiliz* or exercis* or motor activit* or walking or ambulat*) OR AB (physical activit* or physical function* or mobility or mobilis* or mobiliz* or exercis* or motor activit* or walking or ambulat*))

AND

TI (barrier* or enabl* or facilitat* or motivat*) OR AB (barrier* or enabl* or facilitat* or motivat*)

NOT

(((MH "Animals+") OR (MH "Models, Biological") OR (MH "Fungi+") OR (MH "Invertebrates+") OR (MH "Plants+")) OR (TI (animal experiment* or animal model*) OR AB (animal experiment* or animal model*))) NOT (MH "Human")
