# Supplementary material for: Barriers and enablers to physical activity in patients during hospital stay: a scoping review
Source: Syst Rev. 2021 Nov 4;10:293. doi: 10.1186/s13643-021-01843-x (PMC8569983; doi:10.1186/s13643-021-01843-x)
Supplement: Supplementary file 3 — Additional file 3. The Theoretical Domains Framework with definitions and component constructs. Description of the definitions and component constructs of each TDF domain. [file 13643_2021_1843_MOESM3_ESM.docx]

**Additional File 3.** The Theoretical Domains Framework (TDF) with definitions and component constructs

| **TDF domain** | **TDF domain** | **Component constructs** |
| --- | --- | --- |
| **1. Knowledge** | An awareness of the existence of something | Knowledge (including knowledge of conditions/scientific rationale)  Procedural knowledge  Knowledge of task environment |
| **2. Skills** | An ability or proficiency acquired through practice | Skills  Skills development  Competence  Ability  Interpersonal skills  Practice  Skill assessment |
| **3. Social/Professional Role and Identity** | A coherent set of behaviors and displayed personal qualities of an individual in a social or work setting | Professional identity  Professional role  Social identity  Identity  Professional boundaries  Professional confidence  Group identity  Leadership  Organizational commitment |
| **4. Beliefs about Capabilities** | Acceptance of the truth, reality or validity about an ability, talent or facility that a person can put to constructive use | Self-confidence  Perceived competence  Self-efficacy  Perceived behavioral control  Beliefs  Self-esteem  Empowerment  Professional confidence |
| **5. Optimism** | The confidence that things will happen for the best or that desired goals will be attained | Optimism  Pessimism  Unrealistic optimism  Identity |
| **6. Beliefs about Consequences** | Acceptance of the truth, reality or validity about outcomes of a behavior in a given situation | Beliefs  Outcome expectancies  Characteristics of outcome  expectancies  Anticipated regret  Consequents |
| **7. Reinforcement** | Increasing the probability of a response by arranging a dependent relationship, or contingency, between the response and a given stimulus | Rewards (proximal/distal, valued/not  valued, probable/improbable)  Incentives  Punishment  Consequents  Reinforcement  Contingencies  Sanctions |
| **8. Intentions** | A conscious decision to perform a behavior or a resolve to act in a certain way | Stability of intentions  Stages of change model  Trans-theoretical model and stages of change |
| **9. Goals** | Mental representations of outcomes or end states that an individual wants to achieve | Goals (distal/proximal)  Goal priority  Goal/target setting  Goals (autonomous/controlled)  Action planning  Implementation intention |
| **10. Memory, Attention and Decision Process** | The ability to retain information, focus selectively on aspects of the environment and choose between two or more alternatives | Memory  Attention  Attention control  Decision making  Cognitive overload/tiredness |
| **11. Environmental Context & Resources** | Any circumstance of a person’s situation or environment that discourages or encourages the development of skills and abilities, independence, social competence and adaptive behavior | Environmental stressors  Resources/material resources  Organizational culture/climate  Salient events/critical incidents  Person × environment interaction  Barriers and facilitators |
| **12. Social Influences** | Those interpersonal processes that can cause individuals to change their thoughts, feelings or behaviors | Social pressure  Social norms  Group conformity  Social comparisons  Group norms  Social support  Power  Intergroup conflict  Alienation  Group identity  Modelling |
| **13. Emotion** | A complex reaction pattern, involving experiential, behavioral, and physiological elements, by which the individual attempts to deal with a personally significant matter or event | Fear  Anxiety  Affect  Stress  Depression  Positive/negative affect  Burn-out |
| **14. Behavioral Regulation** | Anything aimed at managing or changing objectively observed or measured actions. | Self-monitoring  Breaking habit  Action planning |

TDF = Theoretical Domains Framework

Reference:

Atkins L, Francis J, Islam R, O'Connor D, Patey A, Ivers N, et al. A guide to using the Theoretical Domains Framework of behaviour change to investigate implementation problems. Implement Sci. 2017;12(1):77
