# Supplementary material for: Barriers and enablers to physical activity in patients during hospital stay: a scoping review
Source: Syst Rev. 2021 Nov 4;10:293. doi: 10.1186/s13643-021-01843-x (PMC8569983; doi:10.1186/s13643-021-01843-x)
Supplement: Supplementary file 4 — Additional file 4. Characteristics of included studies. Table presenting the characteristics of included studies. [file 13643_2021_1843_MOESM4_ESM.docx]

**Additional File 4.** Characteristics of included studies

|  | **Type of study** | **Study aim** | **Method** | **Reported by patients or HCPs** | **Population/Setting** | **Study sample** |
| --- | --- | --- | --- | --- | --- | --- |
| Alawadi et al, 2016 [45] | Qualitative | To identify local barriers and facilitators before the adoption of an ERAS pathway for patients undergoing colorectal operations at a safety-net hospital. | Semi-structured interviews | Patients, HCPs | Adult patients who had undergone major colorectal surgery in a safety-net hospital in the USA, and their HCPs (i.e., general surgeons, anaesthesia staff, and perioperative nursing staff). | n=18 patients, n=8 anaesthesiologists, n=5 general surgeons, and n=6 nurses |
| Andreasen et al, 2018 [46] | Qualitative | To identify factors affecting the adherence of patients and staff to an integrated physical activity and nutritional intervention on a medical ward. | Focus group interviews, semi-structured interviews | Patients, HCPs | Adult patients admitted to a medical ward specializing in infectious diseases in a Danish university hospital, and their nursing staff. | n=7 patients, n=5 nursing staff members in n=2 focus groups and n=3 interviews |
| Babine et al, 2019 [47] | Mixed-methods | To explore and describe the characteristics of the Hospital Elder Life Program (HELP) sites and how they mobilize patients with volunteers in the United States and other countries. | Surveys | HCPs | Settings in 25 international locations that were identified using the official HELP site registrant list (i.e., USA, Australia, Austria, Brazil, Canada, China, Columbia, Germany, Hong Kong, India, Israel, Italy, Japan, Mexico, Netherlands, New Zealand, Peru, Poland, Portugal, Republic of Korea, Scotland, Spain, Switzerland, Taiwan, and United Kingdom) | n=28 sites |
| Boltz et al, 2010 [20] | Qualitative | To illuminate the factors that influence the physical function of hospitalized older adults. | Focus group interviews | Patients | Patients aged 70 years and older admitted to three senior centres in the USA and having experienced hospitalization within the past year | n=24 patients in n=3 focus groups |
| Boltz et al, 2011 [16] | Qualitative | To identify nursing staff perceptions of physical function in hospitalized older adults. | Focus group interviews | HCPs | Nursing staff of a suburban community hospital and an urban teaching hospital in the USA | n=43 registered nurses and n=12 patient care associates in n=3 focus groups |
| Boltz et al, 2014 [48] | Mixed-methods | To describe fear of falling and its relationship with physical activity and function in hospitalized older adults. | Chart extraction, observation and interviews | Patients | Patients aged 70 years and older admitted to two medical or medical/surgical units of an urban hospital in the USA | n=41 patients |
| Bradley et al, 2019 [49] | Qualitative | To explore how occupational therapists and physiotherapists constructed and interpreted the meaning of rehabilitation in relation to older people in acute care. | Observations and interviews | Patients, HCPs | Patients aged 65 years and older admitted to an acute medical ward in a general hospital in the UK, and their HCPs (i.e., occupational therapists and physiotherapists) | n=5 patients in observations; n=3 occupational therapists and n=2 physiotherapists in interviews |
| Brown et al, 2007 [15] | Qualitative | To identify barriers to mobility during hospitalization from the perspectives of older patients and their primary nurses and physicians, to compare and contrast the perceived barriers among these groups, and to make a conceptual model. | Interviews | Patients, HCPs | Patients aged 75 years and older admitted to medical wards of a university hospital in the USA and their HCPs (i.e., primary nurses and resident physicians) | n=10 patients, n=10 nurses, and n= 9 physicians |
| Chan et al, 2019 [50] | Qualitative | To describe nurses’ perceptions of facilitators and barriers to hospitalized older patients’ physical activity participation in an acute Asian setting. | Semi-structured focus group interviews | HCPs | Nurses working on various general wards in a hospital in Singapore | n=30 nurses in n=5 focus group interviews |
| Cheah et al, 2011 [83] | Qualitative | To examine older people’s experience of acute hospitalization. | Semi-structured interviews | Patients | Patients aged 65 years and older admitted to an acute-care teaching hospital in Australia | n=6 patients |
| Clarke et al, 2017 [51] | Qualitative | To gain a deeper understanding of how older people spent their time on hospital wards and the impact this had on their feelings of wellbeing. | Interviews | Patients | Patients aged 60 years and older admitted to four medical and two orthopaedic wards in a large general hospital in the UK | n=18 patients |
| Cook et al, 2009 [52] | Qualitative | To understand the barriers to, and facilitators of, optimal thromboprophylaxis. | Interviews | HCPs | Nurses, pharmacists, physicians, managers, hospital administrators, medical residents, and members of the hospitals’ quality improvement team in one university-affiliated and two community hospitals in Canada | n=15 nurses, n=6 pharmacists, n=12 physicians, and n=3 hospital administrators. |
| Dahlke et al, 2015 [53] | Qualitative | To explore nursing practice with hospitalized older adults. | Observations and interviews | HCPs | Nurses and patient care aides working in a specialty geriatric unit in a large teaching hospital and a general medical unit in a smaller community teaching hospital in Canada | n=18 registered nurses, n=3 licensed practical nurses, and n=3 patient care aides;  ▪ Participant observations in n=20;  ▪ Interviews in n= 23 (n=12 were interviewed twice) |
| Dermody et al, 2017 [54] | Qualitative | To examine nurses’ barriers, including knowledge, attitude, and external barriers, to promoting physical activity in hospitalized older adults. | Surveys | HCPs | Nurses working at a variety of non-intensive care units - including neurology, cardiac, pulmonary, nephrology, oncology, and general medical-surgical - in two community-based hospitals in the USA | n=85 nurses |
| Doherty-King et al, 2011 [17] | Qualitative | To explore how nurses make decisions about ambulating hospitalized older adults. | In-depth interviews | HCPs | Nurses working at adult medical or surgical units in two teaching hospitals in the USA | n=25 registered nurses |
| Doherty-King et al, 2013 [76] | Qualitative | To explore the relationship between nurses’ attributions of responsibility for ambulating hospitalized patients and their decisions about whether to ambulate. | In-depth interviews | HCPs | Nurses working at adult medical or surgical unit in two teaching hospitals in the USA | n=25 registered nurses |
| Douglas et al, 2004 [78] | Qualitative | To investigate the perceptions and attitudes of patients to the built environments of NHS Trust hospitals, in order to inform design excellence so as to make future hospitals places and spaces responsive to patient needs. | Semi-structured interviews | Patients | Adult patients admitted to four wards (i.e., surgery, medicine, care of the elderly and maternity) in a hospital in the UK | n=50 patients |
| Douglas et al, 2005 [77] | Mixed-methods | To explore patients’ perceptions of health-care built environments, to assess how they perceived health-care built facilities and designs. To develop a set of patient-centred indicators by which to appraise future health-care designs. | Group conferencing, auto-photographic study, expert exchanges and a survey | Patients | Adults patients admitted to a hospital in the UK | ▪ Autographic study: n=35 patients;  ▪ n=40 novices and an unspecified number of experts (i.e., both patients) in n=8 focus group interviews;  ▪ Survey: n=785 patients |
| Eastham et al, 2016 [55] | Mixed-methods | To explore the design features of a “dementia friendly” acute ward environment and, staff views on the implications of daily activity engagement for patients with dementia. | Research-centred environmental assessment tool and semi-structured survey | HCPs | Staff members of the multidisciplinary team working at an acute ward for patients with dementia in a hospital in the UK | n=2 occupational therapists, n=2 nurses, n=2 physiotherapists and n=2 occupational therapy assistants |
| Eijck et al, 2016 [43] | Mixed-methods | To identify barriers, facilitators and influencing factors to implementation of Function Focused Care in current daily nursing care for stroke patients and geriatric patients aged 65 years and older admitted to a hospital who needed help with mobility, bathing and/or dressing. | Structured observations, a survey and focus groups | HCPs | Nurses working at neurology and geriatric wards of an academic and a non-academic hospital in the Netherlands | ▪ Observations of n=12 nurses  ▪ Survey: n=65 nurses  ▪ n=14 nurses in n=3 focus group interviews |
| Gillis et al, 2008 [82] | Quantitative | To examines nurses’ knowledge, beliefs, attitudes, and confidence regarding providing care to prevent and treat deconditioning in hospitalized older adults. | Descriptive surveys | HCPs | Registered nurses enrolled in the introductory theory courses of a post-registered nurse, bachelor of science in nursing distance program offered by an undergraduate Canadian university | n=157 registered nurses |
| Gordge et al, 2009 [56] | Mixed-methods | To improve practice in the care of the older person to ensure their functional status has not declined at the point of discharge. Specific objectives included: determining available standards of care in relation to assessment of functional ability in the elderly; measuring current practice against standards of care based on the best available evidence; establishing priority areas for practice/improvement; implementing the practice/improvement plan; measuring practice to determine improvement in the management of functional decline in older patients and identifying further areas for investigation. | A range of knowledge translation tools and strategies to develop an intervention, using surveys to evaluate the intervention | Patients, HCPs | Patients aged 45 years and older admitted to medical or orthopaedic wards in a large acute tertiary adult hospital in Australia;  Nurses, occupational therapist, physiotherapist, and geriatrician who participated in the project team of the hospital wards. | n=22 patients and n=9 HCPs (i.e., nurses, occupational therapist, physiotherapist and geriatrician) |
| Graf et al, 2013 [57] | Qualitative | To investigate the factors that influenced the mobilization or lack of mobilization of hospitalized older adults in two medical units. | Semi-structured interviews and observations | HCPs | Nurses and physicians working at an acute care medical unit and a transitional care unit of an academic medical centre located at two distinct sites within a large city in the USA | n=10 registered nurses and n=2 physicians |
| Granger et al, 2018 [42] | Qualitative | To explore physiotherapists’ perceptions regarding barriers and enablers to embedding exercise into routine lung cancer clinical care. | Focus group interviews | HCPs | Physiotherapists working in Australian hospitals in the field of lung cancer | n=8 physiotherapists in n=1 focus group interview |
| Haines et al, 2013 [37] | Quantitative | To measure the incidence of post-operative pulmonary complications (PPC) and risk factors for the diagnosis of PPC, and identify barriers that delayed or prevented mobilization of patients in the early post-operative period after major upper abdominal surgery. | Observations | Patients | Patients of all ages who had undergone major abdominal surgery at one of the two surgical wards in a tertiary hospital in Australia | n=72 patients |
| Holst et al, 2015 [81] | Mixed-methods | To examine how physically active Danish old medical patients are during hospitalization and to achieve knowledge of motivation and barriers to physical activity. | Observations and semi-structured interviews | Patients | Patients aged 60 years and older admitted to two medical departments (haematology and kidney disease) in a Danish university hospital | n=13 patients of which n=10 were interviewed |
| Hoyer et al, 2015 [21] | Quantitative | To develop and conduct a novel multicentre, multidisciplinary survey to assess barriers to early patient mobilization, as perceived by the main providers (nurses and physical and occupational therapists) involved in mobilizing inpatients in the general inpatient medical setting. | Self-administered surveys | HCPs | Nurses and rehabilitation therapists (i.e., physical and occupational therapists) working at four general medicine units (adult inpatient, general care, and nonsurgical medical units) at a quaternary academic hospital and two general medicine units at a community-based hospital in the USA | n=38 rehabilitation therapists, and n=82 nurses |
| Jahn et al, 2012 [36] | Mixed-methods | To explore the application of the Nintendo WiiTM game console to motivate hospitalized adult patients with cancer to be physically active during treatment periods. | Semi-structured interviews | Patients | Adult patients admitted to the department of Radiation Oncology at a German university hospital. | n=7 patients |
| Jonsson et al, 2018 [39] | Quantitative | To describe physical performance and barriers to independent mobilization (on postoperative days one to seven) in patients who underwent acute high-risk abdominal surgery. | Observations and a survey | Patients | Adult patients who had undergone acute high-risk abdominal surgery at a university hospital in Denmark | n=50 patients |
| Juneau et al, 2018 [58] | Mixed-methods | To assess the feasibility of implementing SPecific Retraining in INTerdisciplinarity (SPRINT - an exercise program developed to prevent functional decline during hospitalization of older patients) in the context of a Geriatric Assessment Unit. | Observations, interviews and surveys | Patients, HCPs | Patients aged 80 years and older admitted to a Geriatric Assessment Unit of the ‘Institut Universitaire de Geriatrie de Montreal’, Canada; and their HCPs. | n=19 patients and n= not reported HCPs |
| Kalisch et al, 2006 [75] | Qualitative | To determine nursing care regularly missed on medical-surgical units and reasons for missed care. | Semi-structured focus group interviews | HCPs | Nurses working at medical surgical units in two hospitals in the USA | n=107 registered nurses, n=15 licensed practical nurses, and n=51 nursing assistants in n=25 focus groups |
| Kanaskie et al, 2018 [38] | Qualitative | To explore factors which influence decision-making regarding the use of safe patient handling and mobility technology among registered nurses and nursing assistants. | Focus group interviews | HCPs | Nurses and nursing assistants working at a specialty adult intensive care unit and one medical-surgical unit designated as a bariatric specialty unit of an academic hospital in the USA | ▪ n=14 registered nurses in n=2 focus group interviews;  ▪ n=11 nursing assistants in n=2 focus group interviews |
| King et al, 2016 [59] | Mixed-methods | To develop a system-based intervention including five components that target barriers to nurse-initiated patient ambulation. | Chart extraction and focus group interviews | HCPs | Nursing staff working at a general medical unit in a tertiary academic teaching hospital in the USA | ▪ n=10 registered nurses and n=5 certified nursing assistants participated in n=9 focus group interviews  ▪ n=1 registered nurse participated in a single-person interview |
| de Klein et al, 2019 [19] | Qualitative | To give an overview of factors that influence physical activity of patients by exploring the perspectives of both patients and health-care professionals regarding physical activity during hospital stay. | Semi-structured interviews | Patients, HCPs | Adults patients admitted to a geriatrics or gastroenterology ward at a Dutch university hospital, and their HCPs (nurses, physiotherapists, and physicians) | n=8 patients, n=4 nurses, n=2 physiotherapists, and n=3 physicians |
| Koenders et al, 2018 [44] | Qualitative | To understand beliefs, thoughts, attitudes, and experiences related to physical activity during hospital stay in patients and health care providers. | Semi-structured interviews | Patients, HCPs | Adults patients admitted to the Cardiac Care Unit, Cardiology, or Orthopaedics and Traumatology wards of a Dutch university hospital, and their  HCPs | n=18 patients, n=8 nurses, n=6 nurse assistants, n=6 physical therapists, n=3 physicians, and n=1 physician assistant |
| Kouchel et al, 2017 [60] | Mixed-methods | 1) To assess nursing staff’s perceptions of organizational barriers and facilitators to implementing evidence-based care as it relates to mobility. 2) To assess the extent to which older adults were mobilized in an inpatient medical unit. 3) To assess nursing staff’s knowledge base regarding the importance of mobility among hospitalized older adults. 4) To develop and propose feasible solutions to close performance gaps. | Observations and an online survey | HCPs | HCPs working at an inpatient Acute Care for the Elderly unit - which also provided nursing care for general medicine patients - at a university hospital in the USA | n=14 registered nurses, n=8 clinical support technicians, and n=8 nursing assistants |
| Lafrenière et al, 2017 [79] | Qualitative | To document older adults’ perceptions regarding personal strategies for the prevention of possible functional decline during an episode of hospitalization for an acute illness. | Semi-structured interviews | Patients | Patients aged 75 years and older admitted to two medical units of an acute-care urban university hospital in Canada | n=30 patients |
| Leak Bryant et al, 2017 [61] | Qualitative | To explore perceived exercise benefits and barriers in adults with acute leukaemia who recently completed an inpatient exercise intervention during induction therapy. | Semi-structured interviews | Patients | Adult patients diagnosed with acute leukaemia, receiving induction therapy and admitted to an inpatient haematology/oncology unit at a specialized cancer hospital in the USA | n=6 patients |
| Lee-Steere et al, 2019 [73] | Qualitative | To understand older patients’ perceptions  of delirium and nonpharmacological delirium prevention strategies and their perceptions of barriers and enablers  to involvement in nonpharmacological delirium prevention strategies (e.g. maintaining their nutrition, mobility  and cognitive participation) in the acute care setting. | Semi-structured interviews | Patients | Patients aged 65 years and older admitted to six acute medical, medical specialty or surgical wards in three hospitals in Australia. | n=23 patients |
| Lim et al, 2019 [62] | Mixed-methods | To determine the feasibility and acceptability of a volunteer-led mobility intervention to improve activity levels of older inpatients. | Individual interviews, focus group interviews and observations | Patients, HCPs | Patients aged 70 years and older admitted to an acute medical ward for older people in a hospital in the UK, and their HCPs (i.e., nurses, therapists) and volunteers. | ▪ n=50 patients were recruited before and n=50 patients after the intervention was established  ▪ Individual interviews were performed with n=6 patients  ▪ n=6 volunteers, n=7 therapists and n=6 nurses took part in n=3 focus group interviews |
| Lim et al, 2020 [72] | Qualitative | To explore patients’, their family carers’, and nurses’ perceptions of promotion of mobility among hospitalized older adults. | Semi-structured interviews | Patients, HCPs | Patients aged 65 years and older admitted to a general medical ward of an acute tertiary public hospital in Singapore, and their nurses and family carers. | n=14 patients, n=6 family carers, and n=10 nurses |
| Lyon et al, 2014 [80] | Qualitative | To elicit barriers to implementation and functioning of the ERAS program. | Semi-structured interviews | HCPs | HCPs working at the department of colorectal surgery in a quaternary referral hospital in Australia | n=6 physicians, n=3 nursing unit managers, n=2 nurses, n=1 physiotherapist, n=1 dietician, n=1 clinical nurse specialist, n=1 ERAS coordinator, n=1 care coordinator, n=1 service improvement coordinator |
| Meesters et al, 2019 [63] | Quantitative | To describe the levels of self-reported physical activity during hospitalization and patients’ preferences regarding the promotion of physical activity both in older adult patients and adult patients. | Surveys | Patients | Adult patients admitted to nine nonsurgical wards (i.e., cardiology, endocrinology, lung diseases, gastroenterology, infectious diseases, neurology, nephrology, oncology, and rheumatology) and eight surgical wards (i.e., transplantation and vascular surgery, general surgery and oncology surgery, neurosurgery, orthopaedics, cardiothoracic surgery, trauma surgery, short-stay, and gynaecology) of a university medical centre in the Netherlands | n=345 patients |
| Moore et al, 2014 [18] | Qualitative | To facilitate the spread of the Mobilization of Vulnerable Elders in Ontario (MOVE ON) intervention, the aim of the current study is to develop a mapping guide that links identified barriers and intervention activities to behaviour change theory. | Semi-structured focus group interviews | HCPs | HCPs working at 26 inpatient units in 14 hospitals in Canada, where the MOVE ON intervention was planned to be implemented | n=261 participants in n=46 focus groups across n=26 hospital inpatient units. Participants included nurses, nurse practitioners, occupational therapists, physiotherapists, physicians, managers, various allied health staff members, and other unit staff such as personal support workers and ward clerks. |
| Moreno et al, 2019 [64] | Mixed-methods | To evaluate the impact of advice from a physiotherapist about the importance of staying physically active during hospitalization on activity, mobility, strength, length of stay and complications in older inpatients. In addition, this study also sought to identify the main barriers to staying physically active during hospitalization. | Surveys | Patients | Patients aged 60 years and older admitted to a university hospital ward in Brazil | n=58 patients |
| Mudge et al, 2015 [11] | Mixed-methods | To describe Eat Walk Engage, a collaborative care model on a general medical ward. | Using a variety of quantitative and qualitative methods | Patients, HCPs | Patients aged 65 years and older admitted to a general medical ward in a metropolitan, government-funded teaching hospital in Australia, and their  HCPs (i.e., senior nurses, physiotherapists, occupational therapists, social workers, nutritionists, speech therapists, and physicians) | Not reported |
| Murphy et al, 2018 [65] | Mixed-methods | To determine whether patients were willing to receive physical activity advice during an acute hospital admission and if so, what the patients' preferences were for the content, format and delivery of physical activity advice. | Survey and semi-structured interviews | Patients | Patients of all ages admitted to an acute medical unit of a university hospital in the UK | n=12 patients |
| Nesbitt et al, 2012 [66] | Quantitative | To explore the use of two methods of postoperative ambulation of patients recovering from thoracic surgery: standard method for ambulation versus intravenous pole/walker. | Survey | Patients, HCPs | Patients of all ages who had undergone thoracic surgery at a thoracic surgery unit of a university medical centre in the USA, and their nurses | n=39 patients, and n=36 nurses |
| Parke et al, 2010 [84] | Mixed-methods | To illuminate how problems arise between older adults and the hospital environment through examination of the transactions between the two rather than a focus on one or the other. | Semi-structured interviews and observations | Patients, HCPs | Patients, and hospital employees with more than two years’ experience in administrative, quality improvement, and clinical roles, of a community hospital in Canada | n=8 patients, n=3 spouses, and 14 hospital employees |
| Persson et al, 2015 [67] | Qualitative | To illuminate patients’ experiences of being hospitalized in a hospital with a single-bed room design. | Interviews | Patients | Adult patients admitted to a surgical ward in a hospital building with a single-bed room design in Sweden | n=16 patients |
| Rasmussen et al, 2020 [74] | Qualitative | To investigate the perspectives of health professionals on factors that may affect interventions, including physical exercise and nutrition, for frail older people in relation to discharge after acute admission to hospital | Semi-structured focus group interviews | HCPs | HCPs working with frail older people in a Danish university hospital and a municipality*. | n=1 physiotherapist, n=1 occupational therapist, n=1 social- and healthcare assistant, and n=1 nurse in n=1 focus group discussion |
| Rosbergen et al, 2017 [68] | Qualitative | To understand perceptions and experiences of nursing and allied health professionals involved in implementing an enriched environment in an acute stroke unit. | Semi-structured interviews | HCPs | Nurses and allied health professionals working at an acute stroke unit in a regional Australian hospital | n=7 nursing staff, n=2 senior allied health therapists, and n=1 allied health assistant |
| Sepulveda-Pacsi et al, 2016 [69] | Quantitative | To understand nurses' knowledge and perceptions of the importance of patient ambulation in acute care hospital settings. | Self-administered survey | HCPs | Nurses working at 26 acute adult-inpatient units in two urban teaching hospitals in the USA | n=192 nurses |
| So et al, 2012 [14] | Qualitative | To describe expectations of, and perceived motivators and barriers to, in-hospital exercise of hospitalized older adults. | Semi-structured interviews | Patients | Patients aged 65 years and older admitted to general medical and surgical wards in a public hospital in the USA | n=28 patients |
| Tang et al, 2013 [70] | Qualitative | To explore the experiences of inpatients with an acute exacerbation of Chronic Obstructive Pulmonary Disease (COPD), who participated in a very early exercise program while acutely unwell. | Semi-structured interviews | Patients | Adult patients with an acute exacerbation of COPD admitted to an Australian hospital | n=19 patients |
| Zisberg et al, 2018 [71] | Mixed-methods | To demonstrate an adaptation process guided by the Systems Engineering Initiative for Patient Safety (SEIPS) 2.0 model to articulate site-specific, culturally based interventions to improve in-hospital mobility among older adults. | Observations, personal interviews, a survey and focus group interviews | Patients, HCPs | Patients aged 60 years and older admitted to two internal medicine units at an academic medical centre in Israel, and their HCPs (nurses, nurse’s aides, physical therapists, and physicians) | ▪ Survey in n=203 patients;  ▪ Interviews in n=11 interviews HCPs (head nurse of the hospital, deputy nurse, two unit head nurses and their deputies, four physicians (department heads and their deputies), head of physical therapy.  ▪ Survey in n=116 nurses, nurse’s aides, physical therapists, and medical doctors. ▪ n=not reported unit staff in n=4 focus group interviews |

Abbreviations: HCPs = healthcare professionals; USA = United States of America; UK = United Kingdom; ERAS = Enhanced Recovery After Surgery; NHS = National Health Service; * = data from HCPs working in the municipality was omitted from the data extraction
