# Supplementary material for: Barriers and enablers to physical activity in patients during hospital stay: a scoping review
Source: Syst Rev. 2021 Nov 4;10:293. doi: 10.1186/s13643-021-01843-x (PMC8569983; doi:10.1186/s13643-021-01843-x)
Supplement: Supplementary file 6 — Additional file 6. Barriers to physical activity during hospital stay for acute care as reported by patients and healthcare professionals. Table presenting the barriers to physical activity. [file 13643_2021_1843_MOESM6_ESM.docx]

**Additional File 6.** Barriers to physical activity during hospital stay for acute care as reported by patients and healthcare professionals

| **TDF domain** | **Patient-reported barriers to physical activity** | **N (%)** | **HCP-reported barriers to physical activity** | **N (%)** |
| --- | --- | --- | --- | --- |
|  | Total number of barriers | 264 (100%) | Total number of barriers | 415 (100%) |
| **1. Knowledge** | | | | |
| **Barriers** | Lack of knowledge of the importance of physical activity | 3 | **HCPs’ knowledge** |  |
|  | Lack of knowledge of what patients are allowed to do | 1 | Lack of knowledge of patients’ capabilities | 5 |
|  |  |  | Lack of knowledge of how to mobilize patients | 2 |
|  |  |  | Lack of knowledge of the importance of physical activity | 2 |
|  |  |  | Lack of knowledge of dealing with psychosocial needs | 1 |
|  |  |  | Lack of knowledge of what to advise patients | 1 |
|  |  |  | Lack of knowledge of who to refer to physical or occupational therapist | 1 |
|  |  |  | **Patients’ knowledge** |  |
|  |  |  | Lack of knowledge of what patients are allowed to do | 3 |
|  |  |  | Lack of awareness of negative effects of bed rest | 1 |
|  |  | **4 (1.5%)** |  | **16 (3.9%)** |
| **2. Skills** | | | | |
| **Barriers** | Being dependent on others during physical activity | 7 | **Patients’ skills** |  |
|  |  |  | Being dependent on others during physical activity | 6 |
|  |  |  | Needing assistance from more than one HCP | 1 |
|  |  |  | **HCPs’ skills** |  |
|  |  |  | Lack of skills in assessing patients’ abilities | 2 |
|  |  |  | Lack of skills in culture-sensitive approaches | 1 |
|  |  |  | Lack of skills in dealing with psychosocial needs | 1 |
|  |  |  | Lack of skills in obtaining resources needed to enable physical activity | 1 |
|  |  | **7 (2.7%)** |  | **12 (2.9%)** |
| **3. Social / Professional Role & Identity** | | | | |
| **Barriers** | **Patients’ sick role** |  | **Patients’ role** |  |
|  | The idea that patients should remain in bed | 4 | **Patients’ sick role** |  |
|  | The idea that elderly patients don’t need to be active | 3 | The thought that patients should remain in bed | 5 |
|  | Patients acting passive and dependent | 1 | Patients adopting a passive and dependent attitude | 4 |
|  | Personality and character traits | 1 | The idea that older patients don’t need to be active | 3 |
|  | Having received the status – ‘Waiting for a rehabilitation ward’ | 1 | Personality and character traits | 4 |
|  |  |  | Patients having received the status – ‘Waiting for a rehabilitation ward’ | 2 |
|  |  |  | **HCPs’ role** |  |
|  |  |  | **HCPs’ role (in general)** |  |
|  |  |  | Role clarity – ‘Perceived responsibility for encouraging physical activity’ | 4 |
|  |  |  | Not wanting to be responsible for patients falling | 1 |
|  |  |  | Attributing responsibility for physical activity to others | 1 |
|  |  |  | **Nurses’ role** |  |
|  |  |  | Attributing responsibility to other disciplines | 3 |
|  |  |  | Lack of autonomy | 2 |
|  |  |  | Nurses are not always willing to do other HCPs' work | 2 |
|  |  | **10 (3.8%)** |  | **31 (7.5%)** |
| **4. Beliefs about Capabilities** | | | | |
| **Barriers** | Lack of confidence in own capabilities | 4 | **Patients’ capabilities** |  |
|  | Lack of control | 1 | Patients adopting a more dependent attitude than necessary | 2 |
|  | Reflecting on own accomplishments during hospital stay | 1 | **HCPs’ capabilities** |  |
|  |  |  | **Nurses’ capabilities** |  |
|  |  |  | Lack of confidence in encouraging physical activity | 6 |
|  |  |  | Lack of physical strength to mobilize patients | 2 |
|  |  |  | Lack of confidence in assessing patients’ capabilities | 1 |
|  |  |  | Lack of perceived competence to inform patients | 1 |
|  |  | **6 (2.3%)** |  | **12 (2.9%)** |
| **5. Optimism** | | | | |
| **Barriers** | - | **0 (0%)** | - | **0 (0%)** |
| **6. Beliefs about Consequences** | | | | |
| **Barriers** | **Patients’ beliefs** |  | **Patients’ beliefs** |  |
|  | **Negative consequences of physical activity** |  | Believing that physical activity results in negative consequences | 1 |
|  | Believing that physical activity results in increased risk of an injury | 3 | Falling | 5 |
|  | Falling | 7 | Believing that rest is needed for recovery | 1 |
|  | Uncomfortable feelings (dizzy, tired, exhausted and more) | 2 | **HCPs’ beliefs** |  |
|  | Heart attack | 1 | **Negative consequences of physical activity** |  |
|  | Infection | 1 | Believing that physical activity results in increased risk of patient injuries | 3 |
|  | Believing that physical activity results in missing meals and care | 2 | Falling | 9 |
|  | Believing that physical activity results in being missed by HCPs | 1 | Believing that physical activity results in increased risk of injuries to HCP | 4 |
|  | Believing that rest is needed for recovery | 4 | Family believes rest is needed for recovery | 2 |
|  | Not wanting to inconvenience HCPs | 4 | Lack of supporting evidence | 1 |
|  |  | **25 (9.5%)** |  | **27 (6.5%)** |
| **7. Reinforcement** | | | | |
| **Barriers** | ‘No falls policy’ discourages HCPs from promoting physical activity | 4 | ‘No falls policy’ discourages HCPs from promoting physical activity | 3 |
|  |  |  | Lack of accountability regarding promoting / improving physical activity | 2 |
|  |  |  | Lack of pressure to facilitate early discharge | 1 |
|  |  |  | Absence of requirements to report physical activity | 1 |
|  |  | **4 (1.5%)** |  | **7 (1.7%)** |
| **8. Intentions** | | | | |
| **Barriers** | Lack of patients’ motivation to be active | 3 | Lack of patients’ motivation to be active | 10 |
|  |  |  | Lack of HCPs’ motivation to encourage physical activity | 2 |
|  |  | **3 (1.1%)** |  | **12 (2.9%)** |
| **9. Goals** | | | | |
| **Barriers** | Patients not having a goal to be active | 1 | Patients not having a goal to be active | 1 |
|  |  | **1 (0.4%)** |  | **1 (0.2%)** |
| **10. Memory, Attention and Decision Process** | | | | |
| **Barriers** | Physical activity does not have priority for patients | 4 | **Physical activity does not have priority during hospital stay** |  |
|  | Difficulty remembering information and instructions | 1 | Priority of safety over improving physical function | 10 |
|  | Becoming lower priority when waiting for rehabilitation ward | 1 | Prioritization is forced by lack of time or high workload | 12 |
|  | Priority of hospital culture on safety over promoting physical activity | 1 | Priority of rest / medical treatment / documenting over physical activity | 11 |
|  |  |  | Prioritization forced by high patient acuity | 10 |
|  |  |  | Hospital focus on discharge instead of improving mobility | 2 |
|  |  | **7 (2.7%)** |  | **45 (10.8%)** |
| **11. Environmental Context & Resources** | | | | |
| **Barriers** | **Patient-related factors** |  | **Patient-related factors** |  |
|  | **Medical factors** |  | **Medical factors** |  |
|  | Illness | 10 | Comorbidities | 2 |
|  | Symptoms | 1 | Cognitive problems | 5 |
|  | Fatigue | 7 | Delirium | 2 |
|  | Pain | 7 | Dementia | 1 |
|  | Weakness | 6 | Obesity | 2 |
|  | Impaired physical functioning | 4 | Depression | 2 |
|  | Dizziness | 3 | Illness | 6 |
|  | Dyspnoea | 2 | **Symptoms** |  |
|  | Visual problems | 2 | Weakness | 6 |
|  | Stiffness | 1 | Pain | 6 |
|  | Low blood sugar | 1 | Fatigue | 5 |
|  | Lack of concentration | 1 | Poor balance | 2 |
|  | Feeling traumatized | 1 | Hypotension | 1 |
|  | Poor balance | 1 | Dyspnoea | 1 |
|  | Hypotension | 1 | Low platelets | 1 |
|  | Complications | 2 | Ethnicity | 1 |
|  | Comorbidities | 1 | Language barriers | 3 |
|  | **Care processes and organizational characteristics** |  | Pre-admission functional impairment | 1 |
|  | **Daily schedule** |  | Age | 4 |
|  | Busy daily schedule | 7 | **Care processes and organizational characteristics** |  |
|  | Daily schedule lacks attention for rest | 2 | Patients’ busy daily schedule | 2 |
|  | Lacking insight in own daily schedule | 2 | Restrictive medical devices | 9 |
|  | Daily schedules are inflexible and fixed | 1 | Using restraints as fall prevention strategy | 2 |
|  | Less physical activity in evening and weekend due to less assistance | 1 | Inconsistency in promoting physical activity between shifts / units | 3 |
|  | **Influence of length of stay** |  | Need to manage symptoms | 2 |
|  | Long length of stay decreases motivation | 1 | Patients wearing hospital gowns / pyjamas | 2 |
|  | Short length of stay limits time to be active | 1 | Transportation takes place in a bed or in a wheelchair | 2 |
|  | Restricting medical devices | 8 | Availability of too few patients to organize activities for | 1 |
|  | Wearing hospital gowns / pyjamas | 2 | No physical therapist consulted | 1 |
|  | Being nursed in isolation | 2 | Patients being nursed in isolation | 1 |
|  | Bed-centred care: care processes taking place at bedside | 2 | Bed-centred care: care processes taking place at bedside | 1 |
|  | **Waiting** |  | HCP’s overreliance on incontinence material | 2 |
|  | Responsiveness: timely answering to call light | 1 | Patients eating in bed | 1 |
|  | Waiting for physician rounds | 1 | **Communication** |  |
|  | Medication | 1 | Insufficient communication between nurses | 1 |
|  | Prolonged fasting induces weakness and feeling unwell | 1 | Insufficient interdisciplinary communication | 3 |
|  | **Prescribed immobility** |  | **Timing of physical activity** |  |
|  | Medical reasons necessitating bedrest | 1 | Length of stay is often too short | 1 |
|  | **Organizational characteristics** |  | Preparation for discharge starts too late | 1 |
|  | Hospital environment creates lack of autonomy and freedom | 2 | Physical activity does not start until discharge phase | 1 |
|  | A risk-averse staff culture | 1 | Immediate postoperative physical activity is limited due to tethers and pain | 1 |
|  | **Physical environment of the hospital** |  | **Waiting** |  |
|  | Possibility to go outside | 3 | Nurses waiting for physical therapists | 5 |
|  | **Hospital environment** |  | Nurses waiting for physicians’ orders | 2 |
|  | Lack of appropriate wayfinding | 4 | Nurses waiting for risks to change | 1 |
|  | Lack of recreational facilities | 2 | **Prescribed immobility** |  |
|  | Inactivating hospital environment | 1 | Medical reasons necessitating bedrest | 6 |
|  | Lack of space | 1 | Inappropriate bedrest orders | 3 |
|  | **Unit / ward environment** |  | Weight bearing restrictions | 1 |
|  | Cluttered and busy hallways | 5 | **Organizational characteristics** |  |
|  | Lack of places to rest | 2 | Hospital culture does not encourage physical activity | 1 |
|  | Lack of communal dining room | 1 | Hospital culture does not value prevention interventions | 1 |
|  | **Room environment** |  | Hospital culture does not value physical activity | 1 |
|  | Cluttered and small rooms | 5 | Chaotic hospital atmosphere | 1 |
|  | Lack of appropriate lightning | 3 | **Physical environment of the hospital** |  |
|  | **Influence of the bed** |  | **Hospital environment** |  |
|  | Inadequate height of the bed | 2 | Lack of space | 4 |
|  | Inactivating influence of the bed on the patient | 2 | Lack of appropriate wayfinding | 2 |
|  | Lack of rails | 1 | Lack of appropriate lighting | 2 |
|  | Poor views of natural environment | 1 | Inactivating hospital environment | 1 |
|  | **Limited resources** |  | **Unit/ward environment** |  |
|  | Limited availability of equipment | 4 | Small and cluttered hallways | 3 |
|  | Limited availability of mobility aids | 3 | Unsafe environment | 2 |
|  | Limited availability of furniture | 2 | Lack of places to rest | 2 |
|  | Limited availability of exercise bikes | 1 | **Room environment** |  |
|  | Personal belongings not available at the hospital | 1 | Small and cluttered rooms | 4 |
|  | Limited staffing | 3 | Poor views of the natural environment | 2 |
|  | Limited availability of nursing staff | 3 | Inactivating role of the bed | 2 |
|  | Lack of meaningful activities | 5 | TV positioned above the bed | 1 |
|  | Lack of time – high workload HCPs | 5 | Lack of chairs in the room | 1 |
|  |  |  | Inadequate height of the bed and chairs | 1 |
|  |  |  | **Limited resources** |  |
|  |  |  | Limited staffing | 3 |
|  |  |  | Limited availability of HCPs (in general) | 5 |
|  |  |  | Limited staff during the weekend | 3 |
|  |  |  | Limited availability of physical therapy staff | 5 |
|  |  |  | Limited availability of occupational therapy staff | 1 |
|  |  |  | Limited availability of nursing staff | 12 |
|  |  |  | Lack of continuity in nursing staff | 1 |
|  |  |  | Limited staff through the night / weekend | 2 |
|  |  |  | Limited availability of assistive staff | 1 |
|  |  |  | **Lack of time – high workload for HCPs** |  |
|  |  |  | Lack of time – high workload for nurses | 15 |
|  |  |  | Lack of time – high workload for physical therapists | 2 |
|  |  |  | Lack of time – high workload for HCPs | 8 |
|  |  |  | Limited equipment | 4 |
|  |  |  | Limited mobility aids | 5 |
|  |  |  | Nurses have limited access to mobility aids during the weekend | 1 |
|  |  |  | Limited furniture | 4 |
|  |  |  | Lack of funding | 1 |
|  |  |  | Lack of tools to assess and evaluate patients’ capabilities | 2 |
|  |  | **148 (56.1%)** |  | **210 (50.6%)** |
| **12. Social Influences** | | | | |
| **Barriers** | **HCPs** |  | **HCPs (in general)** |  |
|  | **HCPs (in general)** |  | Lack of team work | 3 |
|  | Providing more care than necessary | 5 | Lack of involving family | 1 |
|  | Lack of assistance | 5 | Providing more care than necessary | 1 |
|  | Lack of encouragement | 3 | Lack of encouragement | 1 |
|  | Negative reactions towards physical activity | 2 | **Patient** |  |
|  | Encouraging patients to take it easy | 2 | Refusal | 5 |
|  | Not discussing physical activity with patients | 1 | **Visitors** |  |
|  | **Nurses** |  | Cultural influences | 1 |
|  | Lack of encouragement | 1 | Providing more assistance than necessary | 1 |
|  | Lack of promoting independence in ADL/walking | 2 | Absence of visitors increases workload of nurses | 1 |
|  | Lack of assistance | 2 | Domestic help at home induces passivity during hospital stay | 1 |
|  | Verbal encouragement perceived as negative | 1 |  |  |
|  | Underestimation of patients’ capabilities | 1 |  |  |
|  | **Physical therapists** |  |  |  |
|  | Absence of a physical therapist | 1 |  |  |
|  | Short physical therapy sessions | 1 |  |  |
|  | Lack of providing tailored care | 1 |  |  |
|  | **Physicians** |  |  |  |
|  | Lack of encouragement | 1 |  |  |
|  | Lack of assistance | 1 |  |  |
|  | Visitors | 2 |  |  |
|  |  | **32 (12.1%)** |  | **14 (3.4%)** |
| **13. Emotion** | | | | |
| **Barriers** | **Fear** |  | **HCPs’ emotions** |  |
|  | **Fear of injuries** |  | **Fear** |  |
|  | Fear of falling | 8 | Fear of complaints | 2 |
|  | Fear of infection | 2 | Fear of injuries | 1 |
|  | Fear of a heart attack | 1 | Fear of falling | 7 |
|  | Fear of fainting | 1 | Fear of interfering with equipment, such as iv-lines | 1 |
|  | Fear of inconveniencing staff | 2 | Nurses’ fear of mobilizing patients without physicians’ orders | 1 |
|  | Fear of being missed by HCPs when being active | 1 | Frustration | 1 |
|  | Embarrassment | 1 | **Patients’ emotions** |  |
|  |  |  | Fear of falling | 3 |
|  |  |  | Loneliness | 2 |
|  |  |  | Sadness | 1 |
|  |  |  | Depression | 1 |
|  |  |  | Stress | 1 |
|  |  |  | Embarrassment | 1 |
|  |  | **16 (6.1%)** |  | **22 (5.3%)** |
| **14. Behavioural Regulation** | | | | |
| **Barriers** | Lack of standardized procedures for promoting physical activity | 1 | Lack of standardized procedures for promoting physical activity | 3 |
|  |  |  | Lack of standardization of the amount of physical activity | 1 |
|  |  |  | Lack of education of patients and visitors | 1 |
|  |  |  | Lack of documentation tools | 1 |
|  |  | **1 (0.4%)** |  | **6 (1.5%)** |

**Legend:** TDF = Theoretical Domains Framework; HCP = healthcare professional; ADL = activities of daily living
