# Supplementary material for: Barriers and enablers to physical activity in patients during hospital stay: a scoping review
Source: Syst Rev. 2021 Nov 4;10:293. doi: 10.1186/s13643-021-01843-x (PMC8569983; doi:10.1186/s13643-021-01843-x)
Supplement: Supplementary file 7 — Additional file 7. Enablers to physical activity during a hospital stay for acute care as reported by patients and healthcare professionals. Table presenting the enablers to physical activity. [file 13643_2021_1843_MOESM7_ESM.docx]

**Additional File 7.** Enablers to physical activity during hospital stay for acute care as reported by patients and healthcare professionals

| **TDF domain** | **Patient-reported enablers to physical activity** | **N (%)** | **HCP-reported enablers to physical activity** | **N (%)** |
| --- | --- | --- | --- | --- |
|  | Total number of enablers | 228 (100%) | Total number of enablers | 409 (100%) |
| **1. Knowledge** | | | | |
| **Enablers** | **HCPs’ knowledge** |  | **HCPs’ knowledge** |  |
|  | HCPs’ knowledge of physical activity | 1 | Nurses’ knowledge | 1 |
|  | **Patients’ knowledge** |  | Knowledge of patients’ capabilities | 7 |
|  | Knowledge of the importance of physical activity | 10 | Knowledge of the importance of physical activity | 7 |
|  | Previous experiences with the positive effects of physical activity | 6 | Knowledge of who to refer to physical therapist | 3 |
|  | Experiences from previous hospital admissions | 3 | Knowledge of motivating factors | 1 |
|  | Knowledge of own capabilities | 3 |  |  |
|  | Knowing the discharge date | 1 |  |  |
|  |  | **24 (10.5%)** |  | **19 (4.7%)** |
| **2. Skills** | | | | |
| **Enablers** | **HCPs’ skills** |  | **HCPs’ skills** |  |
|  | HCPs need to have the skills to assist patients | 2 | Having received training on how to mobilize patients | 5 |
|  |  |  | Having the skills to assess patients’ capabilities | 4 |
|  |  |  | Having communication skills | 1 |
|  |  |  | **Patients’ skills** |  |
|  |  |  | Being independent during physical activity | 1 |
|  |  | **2 (0.8%)** |  | **11 (2.7%)** |
| **3. Social / Professional Role & Identity** | | | | |
| **Enablers** | **Patients’ role** |  | **Patients’ role** |  |
|  | Personality and character traits | 3 | Role clarity – Patients’ perceived responsibility to be active | 2 |
|  | **HCPs’ role** |  | Personality and character traits | 1 |
|  | Personality and character traits | 2 | Patient having received the status – ‘Returning home’ | 1 |
|  | Physical therapist is most suitable to improve physical activity | 1 | **HCPs’ role** |  |
|  |  |  | Personality and character traits | 1 |
|  |  |  | **Multidisciplinary team** |  |
|  |  |  | The multidisciplinary team is dedicated to improving physical activity | 7 |
|  |  |  | Perceiving responsibility as a team | 2 |
|  |  |  | **Physicians’ role** |  |
|  |  |  | Physicians’ perceived responsibility to encourage physical activity | 5 |
|  |  |  | **Physical therapists’ role** |  |
|  |  |  | Physical therapists’ perceived responsibility to encourage physical activity | 6 |
|  |  |  | Physical therapists are the most suitable HCPs to improve physical activity | 3 |
|  |  |  | Physical therapists should especially target high-risk subgroups | 1 |
|  |  |  | Physical therapists’ role in education | 2 |
|  |  |  | Physical therapists’ role in decreasing fall risk | 1 |
|  |  |  | **Nurses’ role** |  |
|  |  |  | Nurses’ perceived responsibility to encourage physical activity | 11 |
|  |  |  | Nurses’ need for authority | 1 |
|  |  |  | Nurses promoting patients’ self-efficacy | 2 |
|  |  | **6 (2.6%)** |  | **45 (11%)** |
| **4. Beliefs about Capabilities** | | | | |
| **Enablers** | Perceiving to be in control | 3 | **HCPs’ beliefs** |  |
|  | Perceiving to be capable to mobilize | 3 | **Physical therapists’ beliefs** |  |
|  | Reflecting on own accomplishments during hospital stay | 1 | Perceiving to be capable of encouraging and assisting patients | 1 |
|  |  |  | **Nurses’ beliefs** |  |
|  |  |  | Perceiving to be capable of encouraging and assisting patients | 6 |
|  |  |  | Perceiving that volunteers are competent to encourage physical activity | 1 |
|  |  | **7 (3.1%)** |  | **8 (2.0%)** |
| **5. Optimism** | | | | |
| **Enablers** | Having a positive attitude | 2 | - |  |
|  |  | **2 (0.9%)** |  | **0 (0%)** |
| **6. Beliefs about Consequences** | | | | |
| **Enablers** | Believing that physical activity is needed for recovery | 14 | Believing that physical activity is needed for recovery | 9 |
|  | Believing that inactivity will result in negative health outcomes | 3 | Believing that inactivity results in negative health outcomes | 3 |
|  |  |  | Believing that physical activity is not harmful | 3 |
|  |  | **17 (7.5%)** |  | **15 (3.7%)** |
| **7. Reinforcement** | | | | |
| **Enablers** | - |  | Being accountable for patient’s physical activity level | 2 |
|  |  |  | Creating a nurse-patient social contract to encourage patients | 2 |
|  |  |  | Using incentives to encourage patients | 2 |
|  |  |  | Institutional pressure for early discharge encourages HCPs | 1 |
|  |  |  | Using incentives to encourage HCPs | 1 |
|  |  | **0 (0%)** |  | **8 (2.0%)** |
| **8. Intentions** | | | | |
| **Enablers** | Patients’ motivation to be active | 5 | **HCPs’ motivation** |  |
|  |  |  | Nurses having motivation to improve outcomes | 2 |
|  |  |  | Nurses having motivation to promote physical activity | 4 |
|  |  |  | HCPs’ willingness to adopt new practices | 1 |
|  |  |  | Leaderships’ willingness to invest money in physical activity resources | 1 |
|  |  |  | HCPs’ motivation to improve outcomes | 1 |
|  |  |  | Patients’ motivation to be active | 2 |
|  |  | **5 (2.2%)** |  | **11 (2.7%)** |
| **9. Goals** | | | | |
| **Enablers** | **Having a goal** |  | Preventing complications | 1 |
|  | Preventing physical decline | 6 | Preventing pressure ulcers | 3 |
|  | Preventing boredom | 5 | Preventing pneumonias | 1 |
|  | Preventing symptoms | 3 | Preventing deep vein thrombosis | 1 |
|  | **To experience the positive effects of physical activity** |  | Preventing physical decline | 4 |
|  | Wanting to recover and go home | 9 | Getting patients ready for discharge | 2 |
|  | Wanting to improve morale and well-being | 2 | Preventing boredom | 1 |
|  | Wanting to pass time | 4 | Promoting physical activity enables assessment of functional capabilities | 1 |
|  | Autonomy and involvement in decision-making | 3 | Goal-setting with the patient regarding the amount of physical activity | 1 |
|  |  | **32 (14.0%)** |  | **15 (3.7%)** |
| **10. Memory, Attention and Decision Process** | | | | |
| **Enablers** | - |  | Physical activity receives priority | 2 |
|  |  |  | Work priorities have to be balanced | 1 |
|  |  | **0 (0%)** |  | **3 (0.8%)** |
| **11. Environmental Context & Resources** | | | | |
| **Enablers** | **Patient-related factors** |  | **Patient-related factors** |  |
|  | **Medical factors** |  | **Medical factors** |  |
|  | Recovery / feeling better | 4 | Recovery / feeling better | 2 |
|  | Ignoring symptoms | 1 | Absence of contraindications to physical activity | 1 |
|  | Single / simple pathology | 1 | Absence of functional decline compared to baseline | 1 |
|  | **Care processes and organizational characteristics** |  | Single, simple pathology | 1 |
|  | Receiving tailored, individualized care | 6 | Pre-admission functional capabilities | 3 |
|  | Symptoms have to be managed | 2 | Small / light patients | 1 |
|  | **Daily schedule** |  | Culture | 1 |
|  | Exercise takes place at structured moments | 1 | **Care processes and organizational characteristics** |  |
|  | Balance between physical activity and rest | 2 | **Communication** |  |
|  | Having permission to be active independently | 1 | Regular multidisciplinary communication | 9 |
|  | Diminishing restricting medical devices | 1 | Multidisciplinary rounds | 4 |
|  | Timing of physical activity – discussing physical activity early in the admission period | 1 | Documenting physical activity and functional status | 3 |
|  | **Physical environment of the hospital** |  | **Daily schedule** |  |
|  | **Hospital environment** |  | Daily schedule enables physical activity | 2 |
|  | Availability of leisure facilities | 3 | Daily schedule enables mobilizing patients up to 3 times a day | 2 |
|  | Availability of interactive trails | 1 | Communal meal times | 1 |
|  | Availability of hand rails | 1 | Balance between physical activity and rest | 1 |
|  | Availability of smoking room | 1 | **Eating** |  |
|  | **Unit / ward environment** |  | Availability of a buffet | 2 |
|  | Availability of communal areas | 5 | Eating out of bed | 1 |
|  | Availability of a clutter-free environment | 2 | Availability of physician orders approving mobilization | 8 |
|  | Availability of marked ambulation routes | 1 | Adequate management of symptoms | 4 |
|  | **Room environment** |  | Multidisciplinary collaboration | 3 |
|  | Availability of doors that can be easily opened | 1 | Providing tailored, individualized care | 3 |
|  | Availability of sufficient space | 1 | Diminishing restricting medical devices | 2 |
|  | Availability of chairs with adequate height | 1 | Providing function focused care | 1 |
|  | Layout of the beds enables social interaction | 1 | Timing of physical activity – initiating physical activity in the late postoperative period | 1 |
|  | Being able to go outside | 1 | Using well established referral pathways | 1 |
|  | **Resources** |  | Patients visiting physicians instead of the other way around | 1 |
|  | **Availability of exercise options** |  | **Organizational characteristics** |  |
|  | Availability of group therapy | 3 | Hospital-wide expectations regarding physical activity | 3 |
|  | Availability of supervised, individualized exercise programs | 2 | Unit expectations regarding physical activity | 2 |
|  | Availability of fun interactive games | 1 | **Physical environment of the hospital** |  |
|  | **Staffing** |  | **Hospital environment** |  |
|  | Availability of adequate staff | 1 | Availability of safe walking areas | 1 |
|  | Continuity in staffing | 1 | Suitable lighting and floor | 2 |
|  | **Availability of equipment** |  | Availability of distance markers in hallways | 2 |
|  | Availability of walking aids | 5 | Wayfinding | 2 |
|  | Availability of eHealth | 2 | Availability of sufficient space | 2 |
|  | Availability of exercise bikes | 2 | Availability of interactive trails | 1 |
|  | Availability of meaningful activities | 9 | **Unit / ward environment** |  |
|  | Gaming | 2 | Availability of communal areas | 4 |
|  |  |  | Availability of a dining room | 2 |
|  |  |  | Activity rooms | 2 |
|  |  |  | Points of interest | 2 |
|  |  |  | Availability of therapy rooms | 1 |
|  |  |  | Availability of places to sit in the hallway | 1 |
|  |  |  | Availability of sufficient space | 1 |
|  |  |  | **Room environment** |  |
|  |  |  | Availability of an en-suite toilet | 2 |
|  |  |  | Ability to go outside | 1 |
|  |  |  | **Resources** |  |
|  |  |  | **Availability of exercise options** |  |
|  |  |  | Availability of group therapy | 2 |
|  |  |  | Availability of a walking programme | 1 |
|  |  |  | Availability of an exercise programme | 1 |
|  |  |  | **Staffing** |  |
|  |  |  | Availability of sufficient staffing | 6 |
|  |  |  | Availability of physical therapists | 7 |
|  |  |  | Availability of occupational therapists | 3 |
|  |  |  | Continuity in staffing between shifts | 2 |
|  |  |  | Availability of certified nurse specialists | 1 |
|  |  |  | Availability of volunteers | 1 |
|  |  |  | Availability of sufficient time | 3 |
|  |  |  | Availability of adequate staffing | 3 |
|  |  |  | **Availability of equipment** |  |
|  |  |  | Availability of mobility supporting equipment | 8 |
|  |  |  | HCPs having access to equipment | 1 |
|  |  |  | Availability of suitable furniture | 5 |
|  |  |  | Availability of sufficient and suitable equipment in therapy and activity rooms | 2 |
|  |  |  | Availability of eHealth | 2 |
|  |  |  | Availability of meaningful activities | 5 |
|  |  | **67 (29.4%)** |  | **143 (35.0%)** |
| **12. Social Influences** | | | | |
| **Enablers** | **HCPs** |  | **HCPs** |  |
|  | **HCPs (in general)** |  | **HCPs (in general)** |  |
|  | Providing encouragement | 8 | Multidisciplinary collaboration | 7 |
|  | Providing assistance | 3 | Positive working relationships | 4 |
|  | Discussing physical activity with patients | 1 | Providing encouragement | 4 |
|  | Providing tailored care | 1 | Involving visitors | 2 |
|  | **Nurses** |  | Providing assistance | 2 |
|  | Providing encouragement | 4 | **Physician** |  |
|  | Relational approach | 4 | Encouraging patients to be active | 1 |
|  | Providing assistance | 3 | Encouraging nurses to mobilize patients | 1 |
|  | **Physical therapists** |  | **Nurses** |  |
|  | Providing encouragement | 5 | Providing encouragement | 8 |
|  | Presence of a physical therapist | 4 | Providing assistance | 4 |
|  | Relational approach | 3 | Presence of nurse specialists | 2 |
|  | Providing tailored care | 1 | Discussing physical activity among nurses | 1 |
|  | **Physician** |  | Presence of nurse assistants | 1 |
|  | Providing encouragement | 3 | **Physical therapists** |  |
|  | **Other patients** |  | Presence of physical therapists | 5 |
|  | Seeking social interaction with other patients | 4 | **Occupational therapists** |  |
|  | Providing support and encouragement | 1 | Presence of occupational therapist | 2 |
|  | Volunteers | 3 | Assisting in independence in ADL | 1 |
|  | Encouragement from volunteers | 2 | Encouraging independence in ADL | 1 |
|  | Visitors | 4 | **Leadership** |  |
|  |  |  | Leadership support | 9 |
|  |  |  | Consistent leadership | 1 |
|  |  |  | Leadership enforcement | 1 |
|  |  |  | Volunteers | 4 |
|  |  |  | Visitors | 5 |
|  |  |  | Providing encouragement | 5 |
|  |  |  | Providing assistance | 4 |
|  |  |  | Providing information regarding patients’ capabilities | 1 |
|  |  | **54 (23.7%)** |  | **76 (18.6%)** |
| **13. Emotion** | | | | |
| **Enablers** | - |  | Job satisfaction | 1 |
|  |  | **0 (0%)** |  | **1 (0.3%)** |
| **14. Behavioural Regulation** | | | | |
| **Enablers** | Using a daily schedule | 4 | Providing education | 2 |
|  | Using an exercise programme | 4 | Patient education | 10 |
|  | Providing education | 2 | HCP education | 4 |
|  | Making performance and expectations visible | 1 | Family education | 3 |
|  | Pacing the amount of physical activity | 1 | Using mobility documentation tools | 8 |
|  |  |  | Making performance and expectations visible | 6 |
|  |  |  | Using an exercise program | 5 |
|  |  |  | Using reminders | 3 |
|  |  |  | Creating a habit: incorporating physical activity in routine care | 3 |
|  |  |  | Using protocols | 3 |
|  |  |  | Using a daily schedule | 3 |
|  |  |  | Appointing mobility champions | 2 |
|  |  |  | Using mobility audits | 1 |
|  |  |  | Use of huddles to improve role clarity | 1 |
|  |  | **12 (5.3%)** |  | **54 (13.2%)** |

**Legend:** TDF = Theoretical Domains Framework; HCP = healthcare professional; ADL = activities of daily living
